# Light Responsiveness and Assembly of Arylazopyrazole-Based Surfactants in Neat and Mixed CTAB Micelles

**DOI:** 10.1021/jacsau.2c00453

**Published:** 2022-10-31

**Authors:** Gunjan Tyagi, Jake L. Greenfield, Beatrice E. Jones, William N. Sharratt, Kasim Khan, Dale Seddon, Lorna A. Malone, Nathan Cowieson, Rachel C. Evans, Matthew J. Fuchter, João T. Cabral

**Affiliations:** † Department of Chemical Engineering, Imperial College London, London SW7 2AZ, U.K.; ‡ Institute for Molecular Science and Engineering, Imperial College London, London SW7 2AZ, U.K.; ¶ Molecular Sciences Research Hub, Department of Chemistry, Imperial College London, London W12 0BZ, U.K.; § Department of Materials Science and Metallurgy, University of Cambridge, Cambridge CB3 OFS, U.K.; ∥ Diamond Light Source, Harwell Science and Innovation Campus, Didcot, Oxfordshire OX11 0DE, U.K.; ⊥ Department of Biology, Lund University, 22100 Lund, Sweden

**Keywords:** photosurfactant, CTAB, arylazopyrazole, micelle, SANS, SAXS, liposome

## Abstract

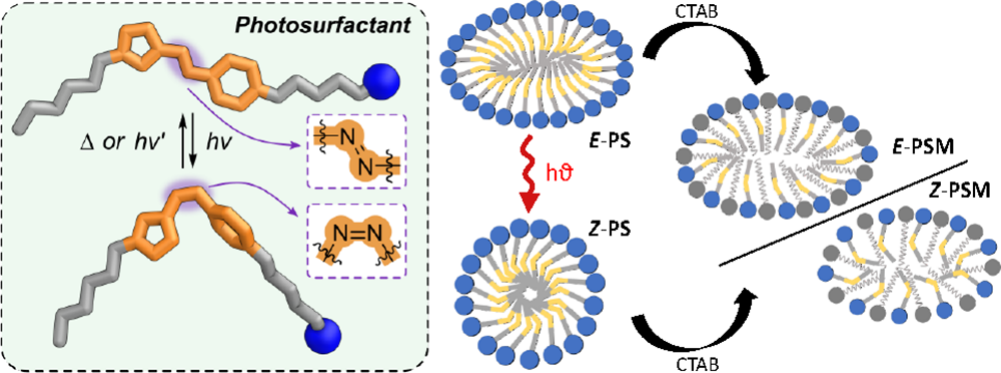

The self-assembly of an arylazopyrazole-based photosurfactant (PS), based on cetyltrimethylammonium bromide (CTAB), and its mixed micelle formation with CTAB in aqueous solution was investigated by small angle neutron and X-ray scattering (SANS/SAXS) and UV–vis absorption spectroscopy. Upon UV light exposure, PS photoisomerizes from *E*-PS (*trans*) to *Z*-PS (*cis*), which transforms oblate ellipsoidal micelles into smaller, spherical micelles with larger shell thickness. Doping PS with CTAB resulted in mixed micelle formation at all stoichiometries and conditions investigated; employing selectively deuterated PS, a monotonic variation in scattering length density and dimensions of the micellar core and shell is observed for all contrasts. The concentration- and irradiance-dependence of the *E* to *Z* configurational transition was established in both neat and mixed micelles. A liposome dye release assay establishes the enhanced efficacy of photosurfactants at membrane disruption, with *E*-PS exhibiting a 4-fold and *Z*-PS a 10-fold increase in fluorescence signal with respect to pure CTAB. Our findings pave the way for external triggering and modulation of the wide range of CTAB-based biomedical and material applications.

## Introduction

The self-assembly and amphiphilic behavior of surfactants is of fundamental importance in diverse fields, including pharmaceutical, food, and cosmetic formulations, biomedical applications, and material synthesis.^1^ In addition to the molecular architecture and electrostatic interactions characteristic of surfactants, numerous strategies are employed to control and modulate the shape and size of micelles, including the addition of cosurfactants and additives or environmental changes (temperature, pressure, pH, etc.). By contrast to these methods, light provides a facile, clean, and noninvasive stimulus to address surfactant systems^2^ as well as allows precise spatiotemporal control over function.^3^ Thus, the allure of light as a preferable alternative for the development of stimulus responsive materials grows commensurately.^2^

Photoswitchable molecules transform between two or more states in response to light and have been employed in a variety of applications, ranging from energy storage^4,5^ to photopharmacology.^6−8^ Of the photoswitches, azobenzenes are arguably the most studied, converting between two isomeric forms *trans* (*E*) and *cis* (*Z*) in response to different wavelengths of light. These isomers display different molecular geometries, electronic absorption spectra, and dipole moments.^9^ Azobenzene motifs have been incorporated into surfactant molecules^10,11^ and studied for their structure–function–assembly relationships.^12−16^ Light-induced switching of a photosurfactant’s conformation^13,17,18^ and properties^19^ has emerged as a powerful strategy for tuning the surfactant’s amphiphilicity on demand.^20^

A subclass of azobenzene photoswitches, the arylazopyrazoles,^21^ have shown near quantitative *E* to *Z* switching,^22^ a long-lived metastable state,^23^ and a large change in the dipole moment.^24−27^ The latter feature has been recently exploited by Kimizuka and co-workers to mediate the solubility of an arylazopyrazole in aqueous media.^24^ These properties facilitate high conversions between the two isomeric forms, while their long-half-lives allow their metastable and assembled structures to be studied and deployed in applications. The arylazopyrazole’s improved photoswitching properties, paired with the greater structural change of the two isomeric forms,^22,23^ render them as ideal candidates to incorporate into photosurfactants.^21,28−30^ Despite showing promise in controlling the assembly of supramolecular structures,^31−36^ relatively few studies have exploited the use of these azoheteroarenes. Specifically, an investigation into the key changes in aggregation behavior occurring between the isomeric states of arylazopyrazoles has not yet been conducted. Moreover, demonstrating the ability to modulate the supramolecular assembly process using light, and characterizing the emerging morphological changes to the system would provide an underpinning framework for applications based on these light-responsive surfactant systems.

**Figure 1:**
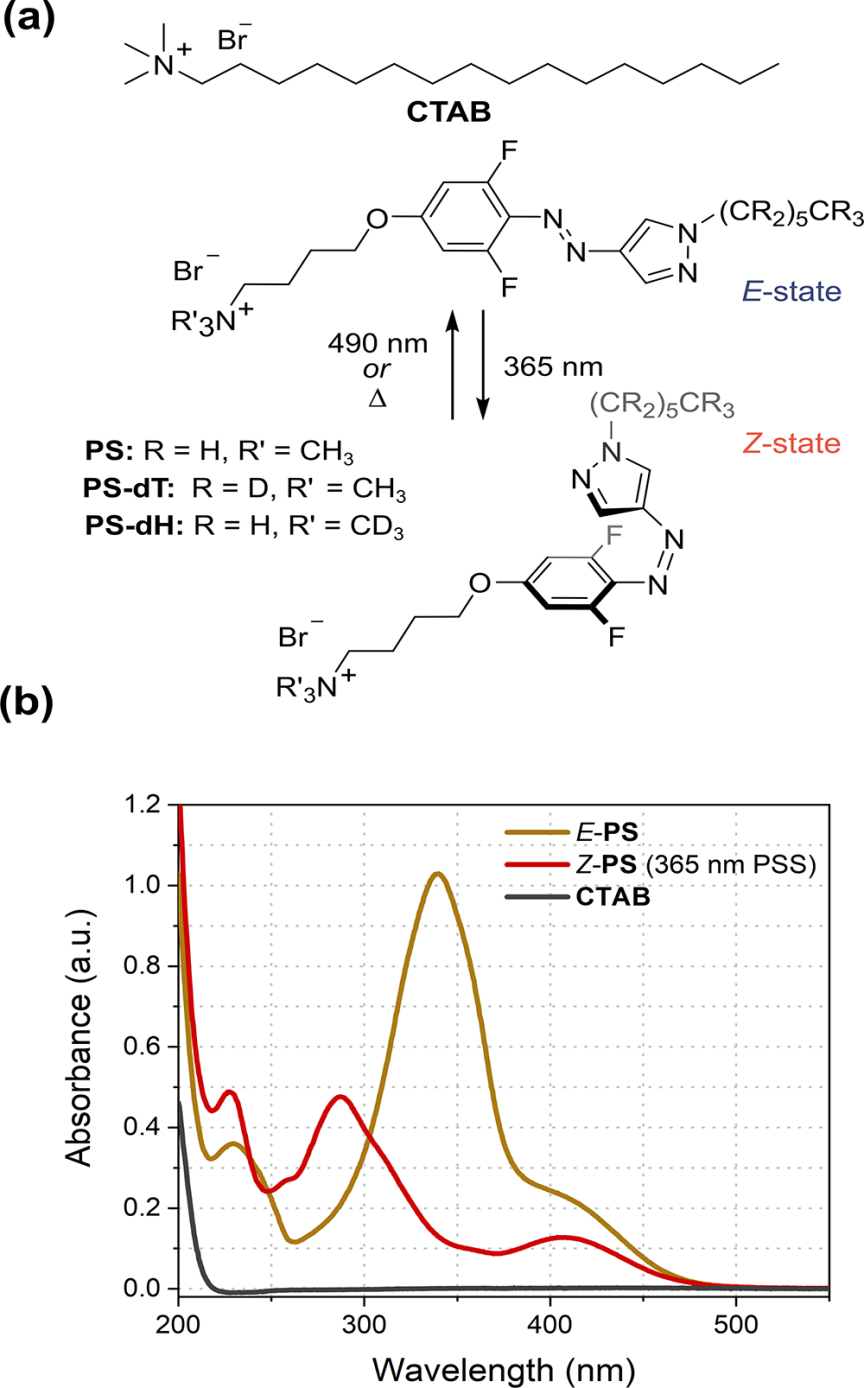
(a) Structure of CTAB and the photoswitchable surfactants investigated. (b) UV–vis absorption spectra of CTAB, *E*-PS, and *Z*-PS measured at 1 mM in H_2_O. *Z*-PS corresponds to the *Z*-rich state achieved by irradiation with 365 nm light.

Herein, we report the synthesis and solution behavior of a water-soluble amphiphilic photosurfactant (PS) that contains an arylazopyrazole motif (Sections 1 and 2, Supporting Information (SI)). The structure of PS was designed to mimic the headgroup and chain length of the ubiquitous cationic surfactant CTAB (cetyltrimethylammonium bromide, Figure 1a). Both the *E* and *Z* isomers of PS were observed to assemble into micelles, and to form mixed micelles with CTAB. Using small-angle neutron and X-ray scattering (SANS and SAXS), we were able to monitor the precise shape and size of neat *E*-PS and *Z*-PS micelles, and mixed micelles with CTAB, as a function of stoichiometry, concentration and illumination.

## Results and Discussion

The synthesis of PS and deuterated analogues employed in our SANS studies (PS-dH and PS-dT) is detailed in Sections 1 and 2 SI. PS was observed to remain dissolved and nonaggregated in DMSO to high concentrations > 10 mM. However, when PS was dissolved in water, the Tyndall effect was observed at high concentrations (>1 mM) attributed to the assembly of PS into micelles. The initial *E*-rich solution of PS was switched to a *Z*-rich photostationary state (PSS) by irradiating with 365 nm light (Figure 1b), achieving 87% of *Z*-PS in water (Figures S2 and S3 SI). The metastable *Z*-state displayed a thermal half-life of 5.7 years at room temperature (25 °C), allowing us to infer that no appreciable thermal *Z* to *E* switching takes place over the course of our measurements (Figure S4 SI). The *E* to *Z* quantum yield of photoisomerization for PS using 365 nm irradiation was measured to be 0.61 (Figure S5 SI).

Combining PS with varying equivalents of CTAB did not yield any noticeable difference in the energy of the electronic absorption bands of the PS. Moreover, no detectable changes in the 365 nm PSS composition were observed for these samples (Figure S6 SI). We determined that the 365 nm PSS of a 1 mM solution of PS could be achieved within 15 min using a lamp of 20 mW/cm^2^, as no further changes could be observed in the UV–vis absorption spectra. Using the same setup, volume, and concentration, subsequent samples were irradiated for 20 min to ensure that the 365 nm PSS was reached. In SAXS experiments employing higher PS concentrations (10–40 mM), the irradiation dose was kept constant and the degree of isomerization tracked by UV–vis absorption spectroscopy (Section 4 SI).

**Figure 2:**
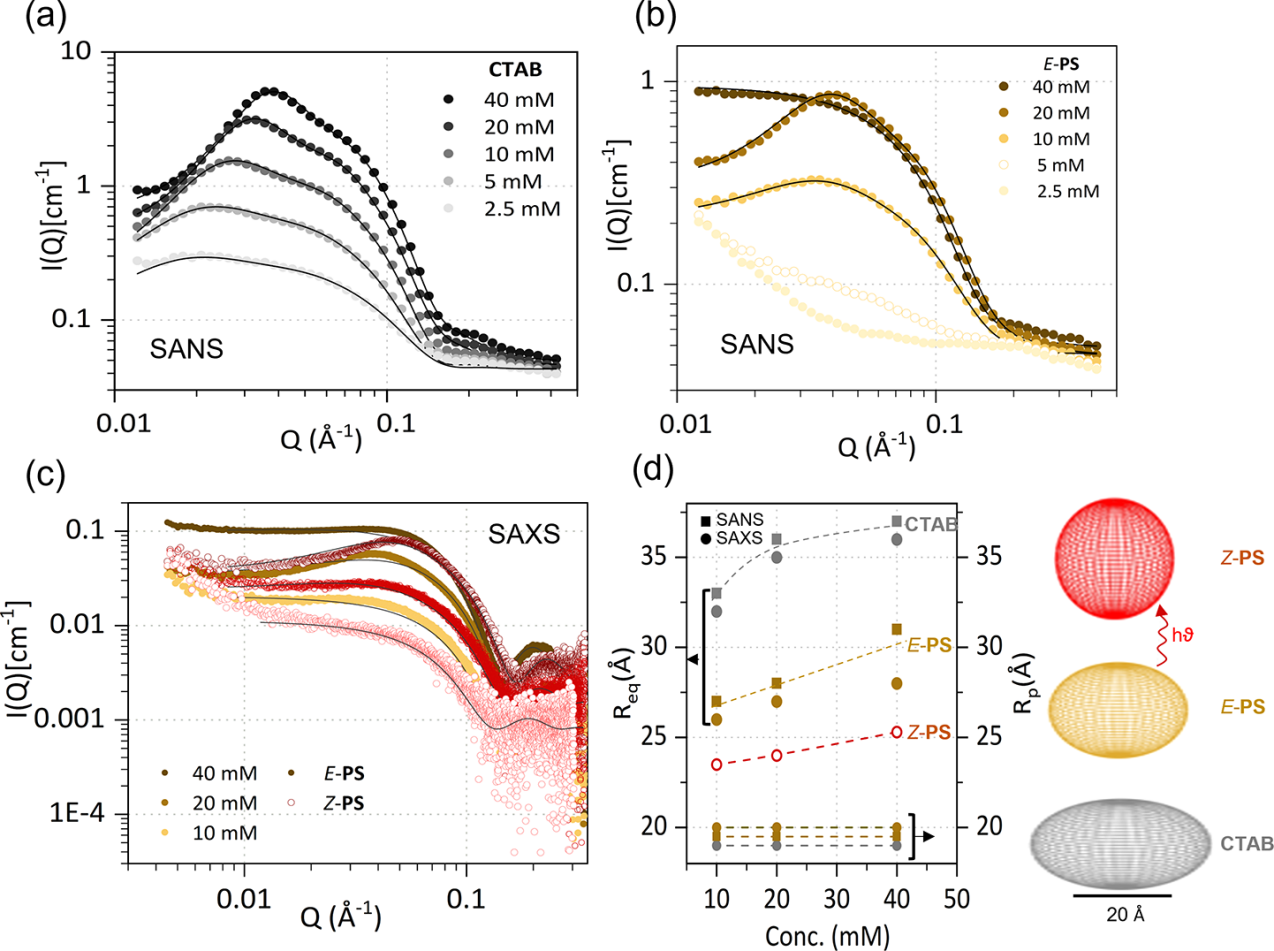
SANS profile of (a) CTAB and (b) *E*-PS in D_2_O at 30 °C at varying concentrations, from 2.5 to 40 mM. Black solid lines correspond to the oblate ellipsoid micellar model. (c) SAXS profile and comparison of scattering intensity before (*E*) and after (*Z*) irradiation with 365 nm light in the concentration range of 10–40 mM. Black solid lines correspond to data fits to oblate ellipsoids in the case of *E*-PS and spherical micelles for *Z*-PS. (d) Micellar radii as a function of concentration for neat CTAB, *E*-PS, and *Z*-PS, where *R*_eq_ and *R*_p_ are the equatorial and polar radii. The representative ellipsoids illustrate the micellar assemblies of CTAB, *Z*-PS, and *E*-PS.

The dimensions, shapes, and interactions of the higher-ordered structures (micelles) formed in solution by *E*-PS, *Z*-PS, and CTAB were determined by a combination of SAXS and SANS. The critical micelle concentration (CMC) for CTAB is ∼1 mM and is estimated to be ∼7 mM for PS from surface tension and SANS experiments. Aqueous solutions in the concentration range 2.5–40 mM were thus chosen for the scattering measurements. Figure 2a and b shows the SANS profile of neat CTAB and *E*-PS micellar solutions as a function of concentration, exhibiting the expected profile for charged micelles, analyzed in terms of form and structure factors. The effect of photoswitching of *E*-PS to *Z*-PS on the micellar size was studied by SAXS, as a function of concentration (10–40 mM) as shown in Figure 2c. The data could be well fitted by a core–shell oblate ellipsoidal form factor, and a mean spherical approximation (RMSA) structure factor, shown by the solid lines. The difference in quality of the fit between oblate and prolate micelles is relatively small, and our analysis follows the detailed study of Bergström and Grillo for CTAB micelles.^37^ The oblate ellipsoids are characterized by an equatorial radius, *R*_eq_ (major) and a polar radius, *R*_p_ (minor): upon increasing the concentration (from 2.5 to 40 mM), *R*_eq_ increases while *R*_p_ remains largely unchanged, indicating micellar growth along the equatorial direction, and these values are in good agreement with previous CTAB reports.^37−39^ The scattering profile of *E*-PS micelles also shows good agreement with an oblate ellipsoidal model for micelles (Figure 2b).

The fitted dimensions of *E*-PS micelles yield a longer minor radius *R*_p_ and shorter major radius *R*_eq_, as compared to neat CTAB. *E*-PS micelles are overall smaller than those of CTAB, despite possessing the same number of carbon atoms in the tail region (Figure 2d). Although the micellar dimensions agree well with the length of the PS determined from quantum chemical studies (Section 3, Figure S1 SI), the overall smaller geometry of PS micelle is somewhat counterintuitive if only the sterics of the two isomers is considered. Specifically, the bulkier arylazopyrazole moiety could result in a larger tail volume, characterized by a larger packing parameter and therefore a larger micelle. We infer that the ability of the photochromic unit of PS to π-stack imposes a tighter packing of the hydrophobic tails, leading to a geometrically constrained hydrophobic region. On the other hand, the aliphatic chain of CTAB exhibits significantly more conformational freedom, thus resulting in a larger micellar size.

SAXS measurements for PS in both the *E* and *Z* states were performed at concentrations above 10 mM, above the CMC of all surfactants. The profiles of *E*-PS micelles were best fitted by oblate core–shell ellipsoidal model and the fitted parameters are in agreement with the values obtained by SANS analysis. Combining SANS and SAXS, the composition of the micellar core and shell were estimated from the respective scattering length densities, and dimensions from independently fitting the same form factor models (Figure 2d). Cryo-TEM imaging also confirms our model fitting of scattering data yielding consistent micellar dimensions for both *E* and *Z*-PS isomers (Figure S7 SI), while SANS/SAXS provides greater discriminating power for the shape and size of the micellar ensembles.

The scattering profiles of *Z*-PS are visibly different and were described instead by a spherical core–shell micelle, indicating an oblate to sphere transition upon *E* to *Z* isomerization of PS (Figure 2d). The transition of oblate ellipsoid for *E*-PS to a spherical micelle in the *Z* state can be attributed to two key changes in the structure of PS: (i) the bent “T-shape” conformation of the *Z*-PS structure prevents the π-stacking possible for *E*-PS^25^ and (ii) an increase in PS’s dipole moment in the tail portion of *Z*-PS.^22,24^

Further, the two isomeric forms of PS exhibit differences in the polar shell, inferred from the SAXS data, namely, an increase in the shell thickness for *Z*-PS. This is consistent with the molecular structure of *E* and *Z*-PS: the charged ammonium headgroup (hydrophilic) is separated from the arylazopyrazole (AAP) unit by a short butyl-chain; AAP possesses a dipole moment in the *E*-state, which increases upon the configurational switch to the *Z*-state. To further probe the difference in micellar polarity, Nile red-loaded micelles were prepared and their fluorescence spectra were measured before and after exposure to light. The decrease in emission intensity observed for *Z*-PS micelles relative to *E*-PS indicates dye release and increased polarity of the former (Figure S9 SI). In addition to a change in the magnitude of the *Z*-PS dipole moment, its orientation also changes. These changes in the dipole moment have the effect of reducing the hydrophobicity of the tail region, and the hydrophilic headgroup can be viewed as extending partially over the AAP unit.

This leads to an increase in the effective headgroup area of the *Z*-PS, a reduction of the packing parameter, and corresponding reduction of equatorial radius (Figure S8 SI). The findings are comparable with the self-assembly behavior of gemini surfactants where headgroup repulsions result in larger separations and give rise to a large effective headgroup area. The resulting packing increases interfacial curvature and results in spherical micelles.^40,41^ The charge per micelle of CTAB is found to be larger than that of either PS micelles, from analysis of the structure factor, as expected given the larger micelle volume and aggregation number of CTAB. Upon illumination, the effective charge of *E*-PS decreases considerably, as a result of the surfactant structural change, which impacts the spatial arrangement of the micellar shell, as well as its shape (Figure S10 SI).

**Figure 3:**
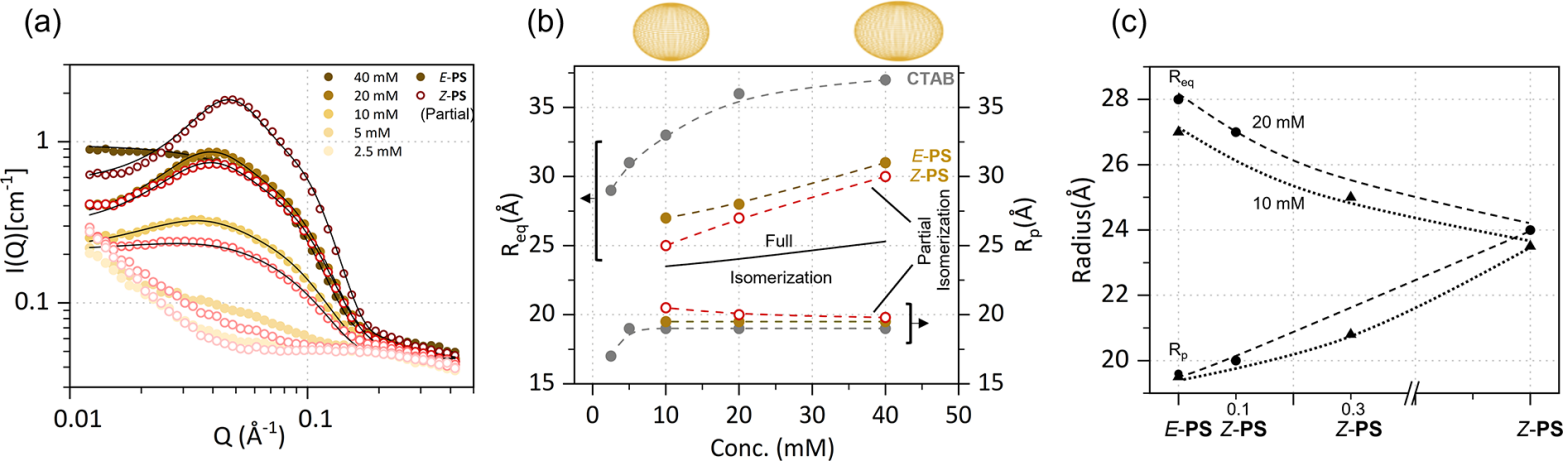
(a) SANS profiles measured for neat *E*-PS at 2.5–40 mM concentrations and subjected to an insufficient light dose, leading to partial conversion to *Z*-PS (detailed in the text). Black solid lines correspond to oblate ellipsoid fits. (b) Micellar equatorial, *R*_eq_, and polar, *R*_p_, radii as a function of surfactant concentration; the black dashed line corresponds to the radius of fully isomerized PS. (c) Radii obtained from fitting the partially isomerized PS samples at selected concentrations of 10 and 20 mM.

Motivated by the practical application of these systems, we examined the effect of *partial* isomerization for samples illuminated with an insufficient UV dose for complete isomerization, at this (10–40 mM) concentration range, viz. 10 min at 20 mW/cm^2^, corresponding to approximately 1/4 of that employed in the previous experiments. The resulting SANS profiles are shown in Figure 3a, and the radii obtained from the oblate ellipsoid model shown in Figure 3b, along with a comparison with the radius in the fully isomerized state. Upon illumination, *R*_eq_ decreases while *R*_p_ increases, albeit modestly (i.e., the micelles evolve toward a spherical shape), as expected for partial isomerization of *E*-PS to *Z*-PS. As seen in Figure 3c, the greater effect of isomerization is reflected in the larger reduction of oblate ellipsoid radii for lower concentrations (10 mM), and thus with greater light transmission.

**Figure 4:**
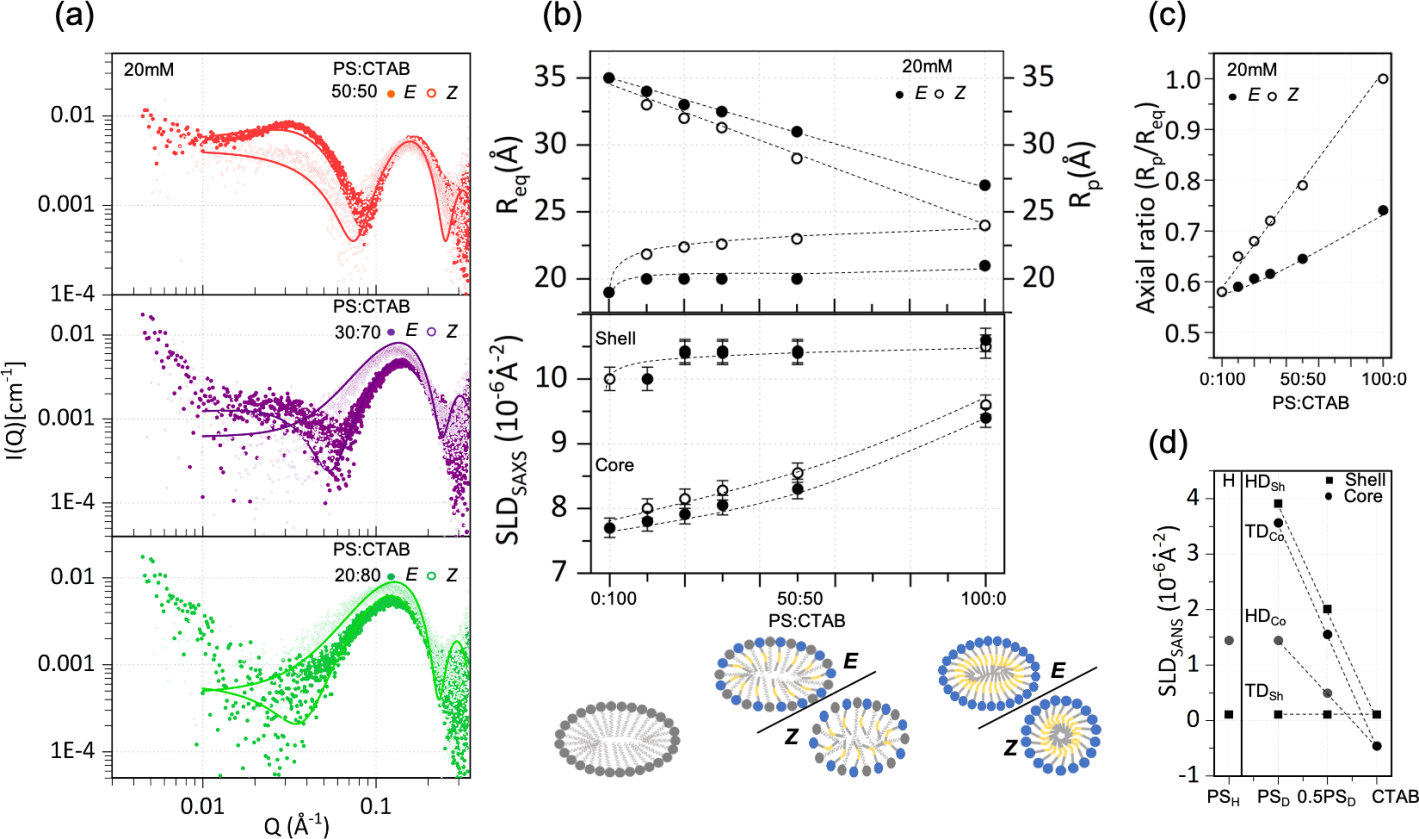
(a) SAXS profiles of PS/CTAB mixed solutions in both *E* and *Z* states at a fixed total concentration of 20 mM at representative molar ratios of PS:CTAB 50:50, 30:70, and 20:80. Black solid lines are fits to oblate ellipsoidal micelles. (b) Micellar equatorial and polar radii, *R*_eq_ and *R*_p_, as a function of surfactant stoichiometry in both *E* and *Z* states (top) and corresponding SLD variation of micellar core and shell obtained from fitting the core–shell oblate ellipsoid model (bottom); the schematics below illustrate the findings. (c) Increase in core aspect ratio of *E*-PS and *Z*-PS as a function of PS/CTAB ratio. (d) Neutron SLD measured for the core (Co) and shell (Sh) of *E*-PS, in hydrogenated (H) and both head (HD) and tail (TD) deuterated forms, in neat and mixed micelles with CTAB.

To probe the feasibility of doping CTAB with neat PS, in terms of mixed micelle formation, a series of mixed surfactant solutions was investigated by SAXS and SANS, benefiting from their complementary contrasts. CTAB is a common, inexpensive surfactant and is used as an antiseptic agent and in a range of biochemical applications. We considered equimolar PS:CTAB solutions down to highly asymmetric ratios with excess CTAB (effectively “doped” by PS). In order to examine the ability to photoswitch the PS *within* CTAB mixed micelles, the mixed solutions were first prepared with the *E*-PS state and subsequently illuminated prior to measurement. Our data show that PS:CTAB mixed micelles are formed at all ratios and with scattering length densities (SLDs) that vary monotonically with stoichiometry. Figure 4a depicts the SAXS profile of representative mixed micelle systems, PSM, in both the *E* and *Z* states. Doping *E*-PS with CTAB resulted in micelles displaying intermediate structural dimensions between CTAB and *E*-PS, as expected for mixed micelles (Figure 4b). In the case of illuminated samples, further reduction in the oblate radii is observed. These results demonstrate that PS is capable of *E*- to *Z*-PS photoisomerization in the presence of CTAB, and the greater the PS stoichiometric ratio, the more spherical the micelles progressively become, as shown in Figure 4c. Moreover, it highlights the significant role that the isomerization state of PS has in defining the geometry of mixed micelles. The increase in SLD is attributed to the gradual increase in electron density, or conversely decrease in molecular volume, from CTAB to *E*-PS to *Z*-PS.

In order to independently resolve the micellar core–shell structure, custom-synthesized selectively deuterated PS surfactant solutions were measured by SANS. These contained either a deuterated tail, PS-dT, or a deuterated charged headgroup, PS-dH. Various contrasts of mixed surfactant solutions were prepared, namely: PS:CTAB, PS-dT:CTAB and PS-dH:CTAB at a 50:50 ratio, and 20 mM total concentration. The SANS and dynamic light scattering (DLS) profiles, detailed in Figures S11 and S12 SI, are characteristic of micelle formation (although some samples exhibit forward scattering, indicative of aggregation induced by interaction changes due to deuteration or the presence of impurities) and the corresponding SLDs are shown in Figure 4d indicating a monotonic trend, corroborating mixed micelle formation in quantitative agreement with the SAXS data. The ability of PS to form mixed micelles and photoisomerize in the presence of CTAB is significant for their practical utilization, given the intrinsic higher cost of PS compared to conventional surfactants.

**Figure 5:**
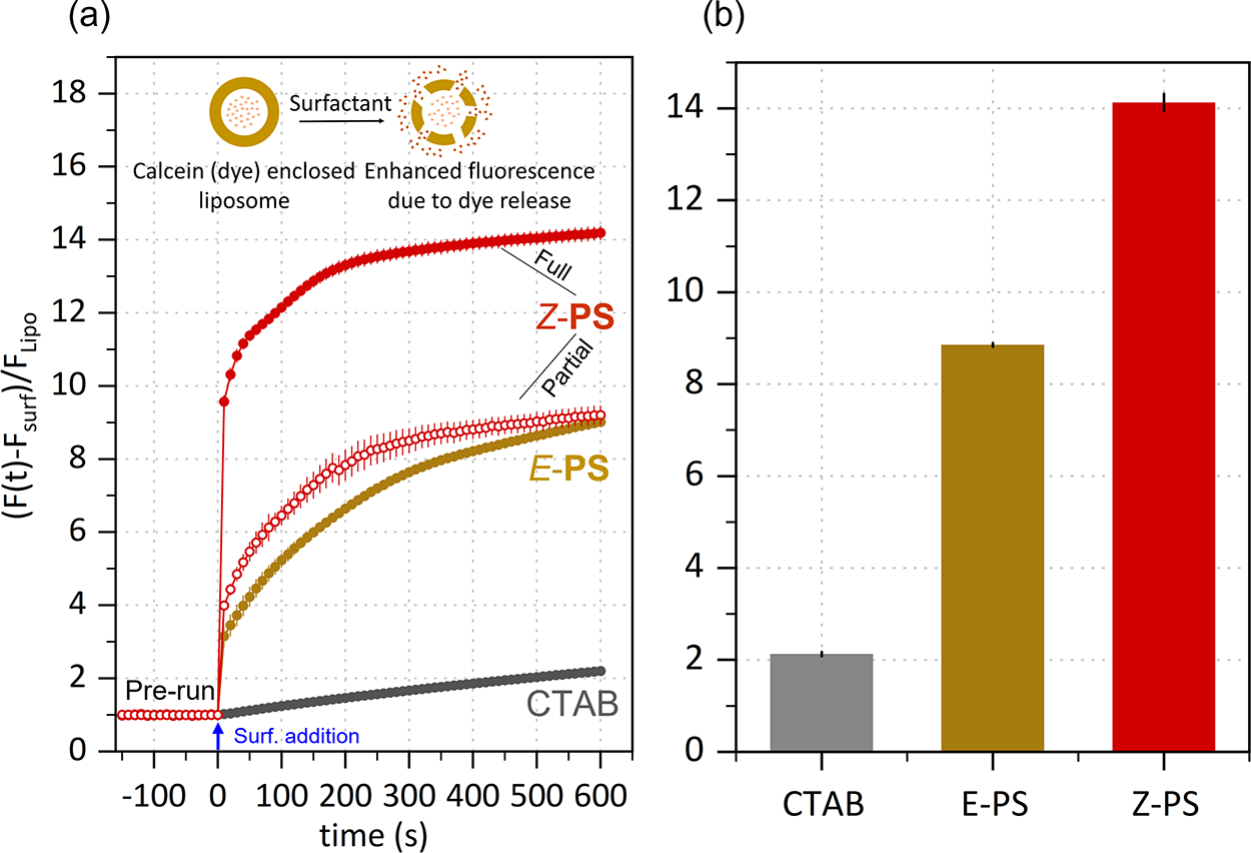
Liposome permeabilization assay: (a) Relative kinetic traces of fluorescence intensity as a result of calcein release from liposomes interacting with surfactants at a concentration of 1 mM, one with full conversion (solid circles) and other with partial conversion (open circles, 1/4 illumination time) of *E*-PS in to *Z*-PS. The fluorescence strength of the dye enclosed liposomes for each run is shown as ’prerun’ while after the addition of surfactant is represented by the expression (*F*(*t*) – *F*_surf_)/*F*_Lipo_, where *F*(*t*) is the integrated fluorescence intensity of reaction at time *t*, *F*_surf_, and *F*_lipo_ are the fluorescence intensities of surfactants and dye-enclosed liposomes, respectively. Each trace is representative of three experimental replicates and their standard deviation is shown as error bars. (b) Quantification of end point (data from the final 100 s of the 10 min experimental run) signal intensities showing a comparative increase in fluorescence of dye from CTAB to *E*-PS and fully converted *Z*-PS.

We next evaluate the photomodulated membrane solubilization activity of *E*-PS and *Z*-PS, referenced to that of CTAB using a liposome permeabilization assay, detailed in Section 5 SI. This cell-free assay measures membrane permeabilization by encapsulating a fluorophore within liposomes and its subsequent release induced by the surfactant-mediated vesicle disruption. This assay has been successfully employed to investigate lipid bilayer leakage induced by intercations with nanoparticle^42^ and domains of viruses.^43^ Calcein (dye) enclosed liposomes were prepared (Figure S13 SI) and their permeabilization was assessed in real time by the increase in fluorescence intensity caused by dequenching of the released dye into the surrounding buffer by the action of surfactants. Figure 5a depicts the normalized fluorescence exhibited by CTAB, *E*-PS, and *Z*-PS at both partial and fully converted state over time. Both *E*-PS and *Z*-PS at 1 mM concentration show a significant increase in dye release compared to pure CTAB and within barely 20 s reaction time, *E*-PS shows an approximately 4-fold increase in fluorescence, while *Z*-PS shows a 10-fold increase, evidencing faster membrane disruption upon light exposure. We interpret this observation in terms of the increased polarity and greater headgroup area of *E*-PS, and further of *Z*-PS, that improves respectively their insertion ability into the liposome bilayer, and their potential to disrupt lipid packing. The superior efficiency of *Z*-PS (Figure 5b) demonstrates promising functionality of these systems in phototriggered antimicrobial formulations.

In summary, we have investigated the photoisomerism and self-assembly of a model CTAB-based photosurfactant with an aryazopyrazole photoswitch, focusing on the impact of illumination on the neat and mixed micelle formation with CTAB. While CTAB and *E*-PS form oblate micelles, photoisomerization into *Z*-PS causes a transition to spherical micelles with lower micellar repulsion. We establish that mixed micelles of PS and CTAB form at all concentrations and stoichiometries investigated, and that *E*-PS undergoes photoisomerization in both neat and mixed micelles. Light exposure increases the shell thickness in both neat PS micelles and, albeit to a lesser degree, doped with CTAB, and the micellar core becomes slightly more compact. In the PS system investigated, the hydrophobic segment of the *Z* isomer is confined primarily to the alkyl tail and that the arylapyrazole (photoswitch) core and the spacer form part of the effective hydrophilic segment of the molecule, and thus photoisomerism can be used to tune the physical properties of the surfactant. Liposome permeabilization (assessed by real-time fluorescence measurement) shows significant promise, demonstrating that *Z*-PS acts significantly faster with greater yield (10×) than CTAB, exhibiting its superior efficacy as an antiseptic agent. Overall, these findings demonstrate the photoisomerization potential of the investigated PS in both neat and doped conditions and highlight the importance of judiciously designing the PS’s structure for a wide range of optically addressable applications.

## Experimental Section

### Photosurfactant Synthesis

The design and synthesis of the photosurfactant (PS) and head/tail deuterated analogues is described in detail in the SI, alongside with accompanying DFT calculations, performed using Gaussian 16.

### Photoswitching

Photostationary states (PSS) were determined using ^1^H NMR spectroscopy (Figure S2). Briefly, a single set of resonances was observed for the preirradiated samples and assigned to the *E*-isomer. Upon 365 nm irradiation, a new set of resonances appeared corresponding to the *Z*-isomer. Irradiation was performed until no further change in the integration of these two signals was observed, indicating that the PSS was obtained. NMR samples were then diluted, and a UV–vis absorption spectrum was recorded. Comparison of the UV–vis absorption spectrum of the pure *E*-isomer with the spectrum of the 365 nm PSS (along with the PSS population obtained via ^1^H NMR) served as a calibration for the UV–vis absorption experiments shown in this work. UV 365 nm irradiation was achieved using a custom-built irradiation setup using 3 × 800 mW Nichia NCSU276A LEDs.

### Small Angle Neutron Scattering (SANS)

Measurements were carried out on the time-of-flight SANS2D diffractometer at ISIS pulsed neutron source (Oxfordshire, UK), with an incident wavelength range of 2–14 Å^–1^, and wavevector *Q* range of 0.005–1 Å^–1^ achieved by two detectors at 2.4 and 4 m from the sample. Banjo quartz cells of 1 mm path length were used for the measurements. Data were reduced and calibrated using MANTID, radially averaged, and analyzed with SasView (v5.0.4) using an ellipsoid form factor and Hayter mean spherical approximation (HMSA) structure factor. To achieve partial isomerization, samples were irradiated at 365 nm for 20 min and 20 mW cm^–2^.

### Small Angle X-ray Scattering (SAXS)

Measurements were performed at BioSAXS beamline B21, Diamond Light Source (Oxfordshire, UK), at 12.4 keV and a detector distance of 4.014 m, yielding the *Q* range 0.0031–0.34 Å^–1^. Samples were loaded into a 96-well PCR plate and stored at 30 °C before injection into a quartz capillary, held at 30 °C, for measurement. All the samples were moved at 1 μL/s through the beam to avoid beam damage. Samples were irradiated at 365 nm for over 6 h using an LED lightbox operating at ∼6 mW cm^–2^, rotating the samples every hour.

### Liposome Permeabilization Assay

Phosphatidylglycerol (PG) and phosphatidylcholine (PC) lipids were dissolved in chloroform and mixed in a 1:1 molar ratio at 5 mg/mL concentration. The solvent was then evaporated to create a thin film and placed under vacuum for at least 1 h. Phospholipid films were hydrated to a 100 mM aqueous solution of calcein containing NaOH (added to facilitate complete dissolution). The flask was swirled gently for 30 s every 5 min for 1 h to ensure encapsulation of calcein into the core of liposomes. Liposomes were then extruded 21 times through a polycarbonate membrane (200 nm pore size). During preparation, liposomes were maintained above the phase transition temperature of lipids (25 °C is acceptable for 1:1 (mol/mol) PG:PC). Calcein enclosed liposomes were separated from free dye by Sephadex G-50 beads column using 1× PBS buffer. Dynamic light scattering and fluorescence spectroscopy confirmed the formation and size uniformity of liposomes (average diameter of 100 nm, Figure S13). A dye release assay was set up with a total reaction volume of 200 μ L (100 μL of liposomes, 80 μL of PBS buffer, and 20 μL of 10 mM surfactant solution, yielding 1 mM concentration in the reaction mixture). Assay components were added directly to the wells of a 96-well solid black plate, adding the surfactant last. The reaction volume was gently mixed, and the plate was transferred to a Spectra Max M2 microplate reader with the excitation/emission set to 490/515 nm. Fluorescence data were collected every 10 s for 10 min at room temperature, and normalized.

## Supporting Information Available

NMR and UV–vis spectroscopy characterization data of photosurfactants and isomerization. Structural characterization details of SANS and SAXS measurements and liposome permeabilization assay. Figures depicting the charge, polar shell thickness extracted from fitted scattering data, DLS profiles, cryo-TEM images and characterization of dye enclosed liposomes (PDF)
