# Supplementary material for: Light Responsiveness and Assembly of Arylazopyrazole-Based Surfactants in Neat and Mixed CTAB Micelles
Source: JACS Au. 2022 Oct 31;2(12):2670–7. doi: 10.1021/jacsau.2c00453 (PMC9795462; doi:10.1021/jacsau.2c00453)
Supplement: Supplementary file 1 — au2c00453_si_001.pdf [file au2c00453_si_001.pdf]

Supporting Information

Light responsiveness and assembly of arylazopyrazole-based surfactants in neat and mixed CTAB micelles

Gunjan Tyagi,[a,b,†] Jake L. Greenfield,[b,c,†] Beatrice E. Jones,[d,e] William N. Sharratt,[a] Kasim Khan,[f] Dale Seddon,[a] Lorna A. Malone,[e] Nathan Cowieson,[e] Rachel C. Evans,[d] Matthew J. Fuchter,[b,c*] and João T. Cabral[a,b*]

[a] Department of Chemical Engineering, Imperial College London, London SW7 2AZ, U.K.

[b] Institute for Molecular Science and Engineering, Imperial College London, London SW7 2AZ, U.K.

[c] Molecular Sciences Research Hub, Department of Chemistry, Imperial College London, London W12 0BZ, U.K.

[d] Department of Materials Science and Metallurgy, University of Cambridge, Cambridge CB3 OFS, U.K.

[e] Diamond Light Source, Harwell Science and Innovation Campus, Didcot, Oxfordshire, OX11 0DE, UK.

[f] Department of Biology, Lund University, 221 00 Lund, Sweden.

[†]Authors contributed equally to this work

*Correspondence to: m.fuchter@imperial.ac.uk and j.cabral@imperial.ac.uk

1. Materials and Methods

All reagents and solvents were purchased from commercial suppliers unless specified. Solvents used were dry and of HPLC grade.

UV-Visible measurements were performed on a Cary 60 UV-Vis spectrophotometer, with 10 mm, 0.5 mm path length quartz cuvettes at 295 K, unless otherwise stated. An optically transparent thin layer electrochemical (OTTLE) cell was used without applying potential to achieve a pathlength of 0.19 mm. Solutions for the UV-Vis measurements were made using HPLC grade solvents. A background measurement containing only the solvent used was recorded before measuring samples. This background was subtracted from the sample data using OriginPro (2020, SR1) Software.

High-resolution mass spectra (ESI, APCI) were recorded by the Imperial College London Department of Chemistry Mass Spectroscopy Service using a Micromass Autospec Premier and Micromass LCT Premier spectrometer.

NMR spectra were recorded at 298 K using a Bruker AvanceIII HD Smart Probe 500 MHz spectrometer and a Bruker AvanceIII HD Smart Probe 400 MHz spectrometer, automatically tuned and matched to the correct operating frequencies. TopSpin 3.5 and Mestrenova 8.0.0 S3 were used to apply phase and baseline corrections. ^{1}H and ^{13}C NMR spectra were referenced to the residual solvent peak, and the ^{19}F NMR spectra of organic molecules were referenced to hexafluorobenzene at –164.9 ppm and trifluoroacetic acid at -76.55 ppm. Signals are reported in terms of chemical shift (ppm) and coupling constants (Hz). Abbreviations for multiplicity are as follows: s, singlet; d, doublet; t, triplet; m, multiplet; br, broad; hept, heptet.

365 nm irradiation was achieved using a custom-built irradiation setup. 365 nm was achieved using 3 × 800 mW Nichia NCSU276A LEDs. Ferrioxalate actinometry was used to determine the photon flux from a Nichia NCSU276A 365 nm LED (365 nm, 800 mW @ 100% power) operating at 5% power, fitted with a collimating lens, following a previously reported procedure.[1] The cuvette was placed 3.5 cm away from the light source. The photon flux of the 365 nm LED operating at 5% power for our setup was determined to be 1.845×10^{16} photons/s.

Photo stationary states (PSS) were determined using ^{1}H NMR spectroscopy (Figure S2). Briefly, a single set of resonances were observed for the pre-irradiated samples which were assigned to the *E*-isomer. Upon 365 nm irradiation, a new set of resonances appeared corresponding to the *Z*-isomer. Irradiation was performed again until no further change in the integration of these two sets of signals were obtained, indicating that the PSS was obtained. This NMR samples was then diluted, and a UV-vis spectrum was recorded. Comparison of the UV-vis spectrum of the pure *E* isomer with the spectrum of the 365 nm PSS (along with the PSS population obtained via ^{1}H NMR) served as a calibration for the UV-vis experiments shown in this work.

2. Synthesis and Characterisation

Scheme S1. An overview of the synthetic pathway to obtain photo surfactant (**PS)** and its deuterated analogues, **PS-dH** (head deuterated) and **PS-dT** (tail deuterated).

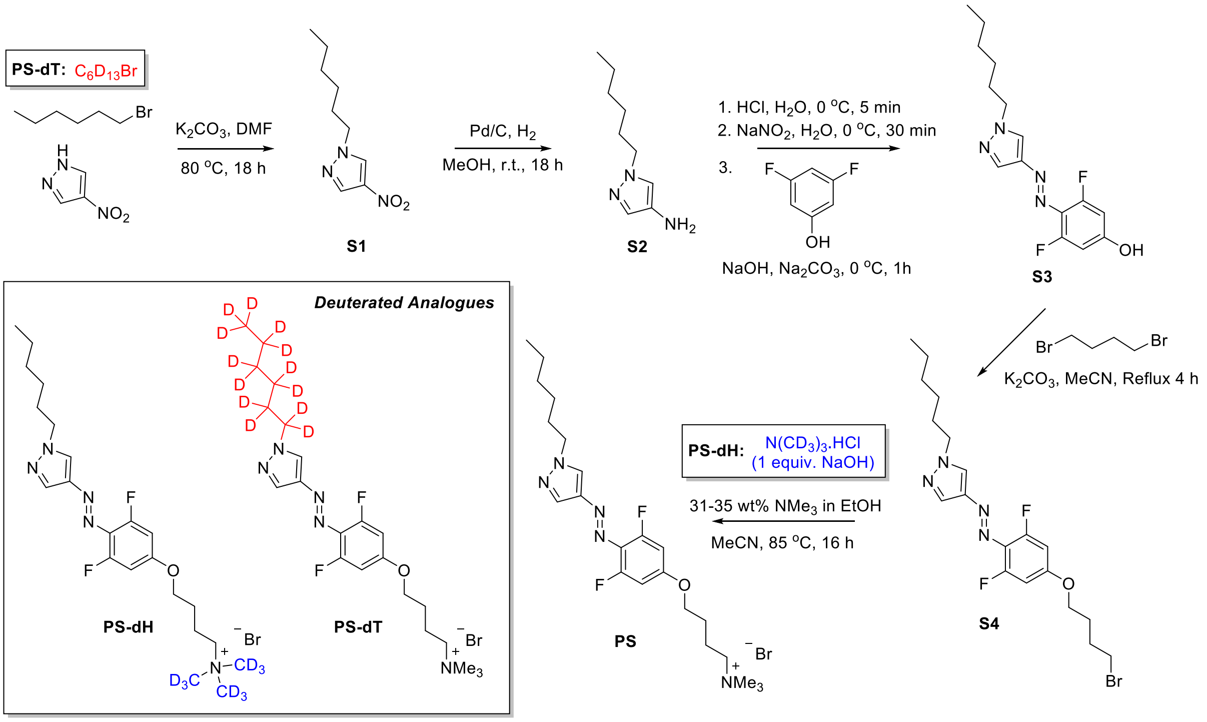

Synthesis of 1-hexyl-4-nitro-1H-pyrazole, S1

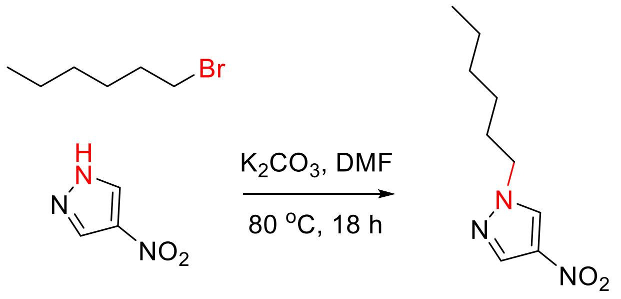

S1

In a flask equipped with a condenser was added 4-nitro-1H-pyrazole (10.0 g, 88.4 mmol, 1 eq), 1-bromohexane (14.84 mL, 0.106 mol, 1.2 eq), K_2CO_3 (24.4 g, 0.177 mol, 2 eq) and 50 mL of DMF. The mixture was heated at 80 °C for 18 h under a N_2 atmosphere. The reaction was cooled to 20 °C and filtered under gravity. The solution was concentrated *in vacuo* and then diluted with 80 mL of EtOAc. The organic phase was washed with 2 × 20 mL of LiCl (aq, 5 w.t.%) and dried over $MgSO_4$. The resulting solution was concentrated *in vacuo* and purified by column chromatography (SiO_2, Hexane:EtOAc, 1:1 to 0:1) to yield **S1** as a colourless oil (14.82 g, 85% yield); ^{1}H NMR (400 MHz, 298 K, $CDCl_3$): δ 8.12 (s, 1H, ***H***-5), 8.00 (s, 1H, ***H***-3), 4.11 (t, *J* = 7.23 Hz, 2H, NC***H***$_2$), 1.89-1.80 (m, 2H, NCH_2C***H***$_2$), 1.29-1.22 (m, 6H, (C***H***$_2$)$_3$$CH_3$), 0.82 (t, *J* = 6.99 Hz, 3H, C***H***$_3$) ppm; ^{13}C NMR (100 MHz, 298 K, $CDCl_3$): δ 135.6, 128.3, 53.5, 31.1, 29.7, 26.0, 22.4, 13.9 ppm; HRMS (APCI, CD_3CN) for [**S1**+H]$^+$: *m/z* calcd: 198.1237; found 198.1241 (Error: 2 PPM).

Synthesis of 1-hexyl-1H-pyrazol-4-amine, S2

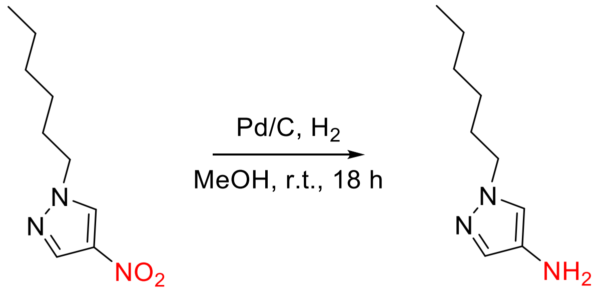

S1 **S2**

Compound **S1** (1.22 g, 6.19 mmol, 1 eq) was dissolved in 40 mL of MeOH. The solution was degassed by bubbling N_2 for 10 min. 10% Pd/C (120 mg, 0.1 eq) was added to the reaction and further degassed by bubbling with N_2 for 5 min. The N_2 was displaced by bubbling H_2 for 10 min. The reaction was left to stir under a positive H_2 atmosphere overnight with vigorous stirring. The mixture was filtered through Celite and concentrated *in vacuo*, yielding an orange oil that as used without further purification, **S2** (0.96 g, 93% yield); ^{1}H NMR (400 MHz, 298 K, $CDCl_3$): δ 7.05 (s, 1H, ***H***-3), 6.91 (s, 1H, ***H***-5), 3.90 (t, *J* = 7.27 Hz, 2H, NC***H***$_2$), 2.87 (br s, 2H, NH_2), 1.76-1.65 (m, 2H, NCH_2C***H***$_2$), 1.27-1.16 (m, 6H, (C***H***$_2$)$_3CH_3$), 0.80 (t, *J* = 6.61 Hz, 3H, C***H***$_3$) ppm; ^{13}C NMR (100 MHz, 298 K, $CDCl_3$): δ 130.7, 128.6, 118.2, 52.2, 31.3, 30.3, 26.2, 22.4, 13.9 ppm; HRMS (ESI, CD_3CN) for [**S2**+H]$^+$: *m/z* calcd: 168.1501; found 168.1497 (Error: 2.4 PPM).

Synthesis of 3,5-difluoro-4-((1-hexyl-1H-pyrazol-4-yl) diazenyl) phenol, S3

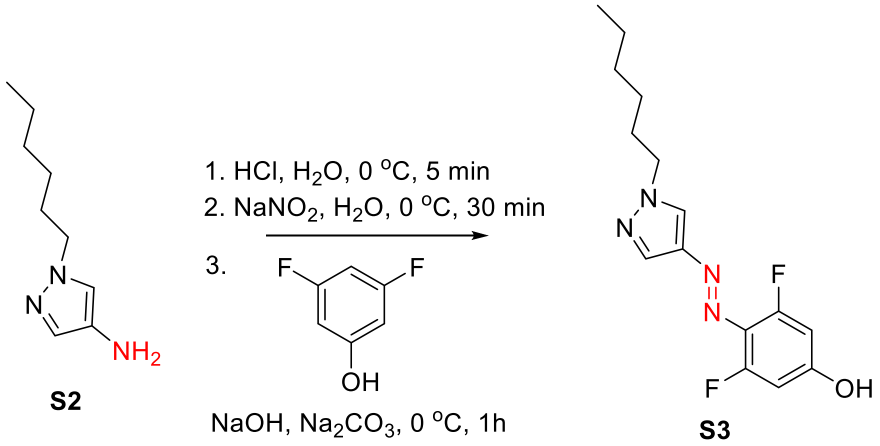

S2 (0.74 g, 4.4 mmol, 1 eq) was dissolved in 20 mL of H_2O and 4.8 mL of HCl (37% in H_2O, 66 mmol, 15 eq) and cooled to 0 °C. To this solution was added a 0 °C solution of $NaNO_2$ (392 mg, 6 mmol, 1.4 eq) dissolved in H_2O (20 mL), dropwise, and was stirred for 30 min at 0 °C. In a separate flask, 3,5-difluorophenol (760 mg, 5.2 mmol, 1.2 eq) and NaOH (1.92 g, 46.4 mmol) was dissolved in 20 mL of water and cooled to 0 °C. This basic solution was added to the pyrazole dropwise, followed by the immediate addition of Na_2CO_3 (4.20 g, 39.6 mmol) in 40 mL of water. The solution was left to stir at 0 °C for 1 hour. The solution was neutralised, which resulted in precipitation of a solid. The solid was collected by suction filtration and washed with H_2O (3 × 30 mL) followed by drying under high vacuum overnight. This afforded a brown solid which was purified by column chromatography (SiO_2, Hexane:EtOAc, 1:1

to 0:1) to yield **S3** as a yellow viscous oil (1.13 g, 83% yield); ^{1}H NMR (400 MHz, 298 K, $CDCl_3$): δ 8.08 (s, 1H, ***H***-5), 8.02 (s, 1H, ***H***-3), 6.55-6.49 (m, 2H, Ar-H), 4.17 (t, *J* = 7.07 Hz, 2H, NC$\boldsymbol{H}_2$), 1.92-1.84 (m, 2H, $NCH_2C\boldsymbol{H}_2$), 1.34-1.25 (m, 6H, $(C\boldsymbol{H}_2)_3CH_3$), 0.85 (t, *J* = 6.93 Hz, 3H, C$\boldsymbol{H}_3$) ppm; ^{13}C NMR (100 MHz, 298 K, $CDCl_3$): δ 159.4, 158.7, 156.2, 142.4, 133.0, 126.1, 124.7, 100.7, 100.4, 53.1, 31.3, 30.2, 26.2, 22.5, 14.0 ppm; $^{19}F\{^{1}H\}$ NMR (378 MHz, 298 K, $CDCl_3$): δ –118.81 ppm; HRMS (ESI, CD_3CN) for [**S3**+H]$^+$: *m/z* calcd: 309.1527; found 309.1520 (Error: 2.3 PPM).

Synthesis of 4-((4-(4-bromobutoxy)-2,6-difluorophenyl)diazenyl)-1-hexyl-1H-pyrazole, S4

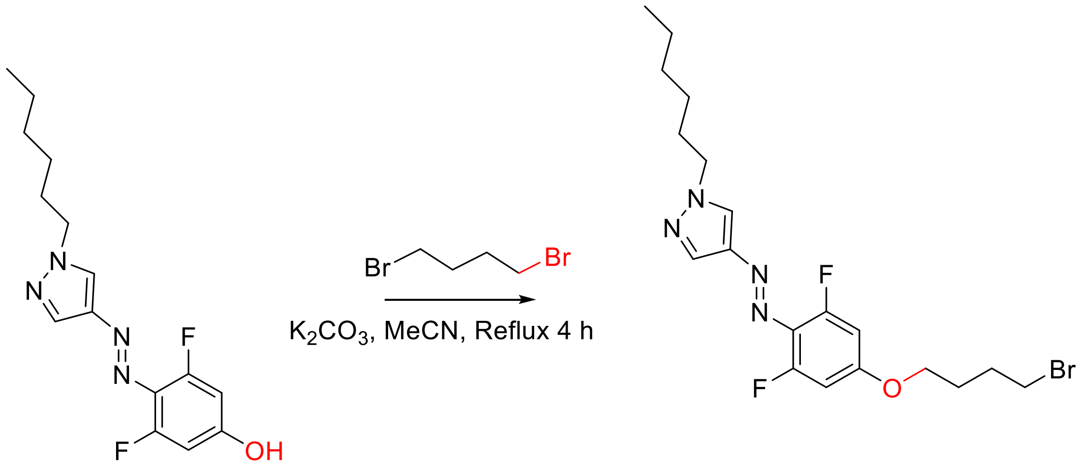

S3 **S4**

To a dried flask was added **S3** (452 mg, 1.5 mmol, 1 eq), 1,4-dibromobutane (0.35 mL, 2.9 mmol, 2 eq), K_2CO_3 (0.83 g, 6 mmol, 4 eq) and dry MeCN (60 mL). The mixture was left to heat under reflux for 4 h. The reaction was quenched by addition of H_2O, resulting in the precipitation of a brown material. The solid was collected by filtration and washed with H_2O (40 mL). The filter cake was dissolved in EtOAc and dried over Na_2SO_4. The solvent was removed under reduced pressure and the resulting brown solid was purified by column chromatography (SiO_2, Hexane:EtOAc, 1:0 to 1:1) yielding **S4** as a yellow solid (0.48 g, 72% yield); ^{1}H NMR (400 MHz, 298 K, $CDCl_3$): δ 7.99 (s, 1H, ***H***-5), 7.97 (s, 1H, ***H***-3), 6.55-6.49 (m, 2H, Ar-H), 4.13 (t, *J* = 7.05 Hz, 2H, NC$\boldsymbol{H}_2$), 3.99 (t, *J* = 5.90 Hz, 2H, OC$\boldsymbol{H}_2$), 3.47 (t, *J* = 6.51 Hz, 2H, C$\boldsymbol{H}_2$Br), 2.08-1.84 (m, 6H, $NCH_2C\boldsymbol{H}_2$ and $OCH_2C\boldsymbol{H}_2C\boldsymbol{H}_2$), 1.36-1.23 (m, 6H, $(C\boldsymbol{H}_2)_3CH_3$), 0.86 (t, *J* = 6.78 Hz, 3H, C$\boldsymbol{H}_3$) ppm; ^{13}C NMR (100 MHz, 298 K, $CDCl_3$): δ 160.0, 158.4, 155.8, 142.5,

133.1, 125.9, 125.4, 99.4, 99.1, 67.9, 53.1, 33.2, 31.3, 30.1, 29.3, 27.6, 26.3, 22.5, 14.1 ppm; $^{19}F\{^1H\}$ NMR (378 MHz, 298 K, $CDCl_3$): δ –119.05 ppm; HRMS (ESI, CD_3CN) for [**S4**+H]$^+$: *m/z* calcd: 443.1258; found 443.1266 (Error: 1.8 PPM).

Synthesis of 4-(3,5-difluoro-4-((1-hexyl-1H-pyrazol-4-yl)diazenyl)phenoxy)-N,N,N-trimethylbutan-1-aminium bromide, PS

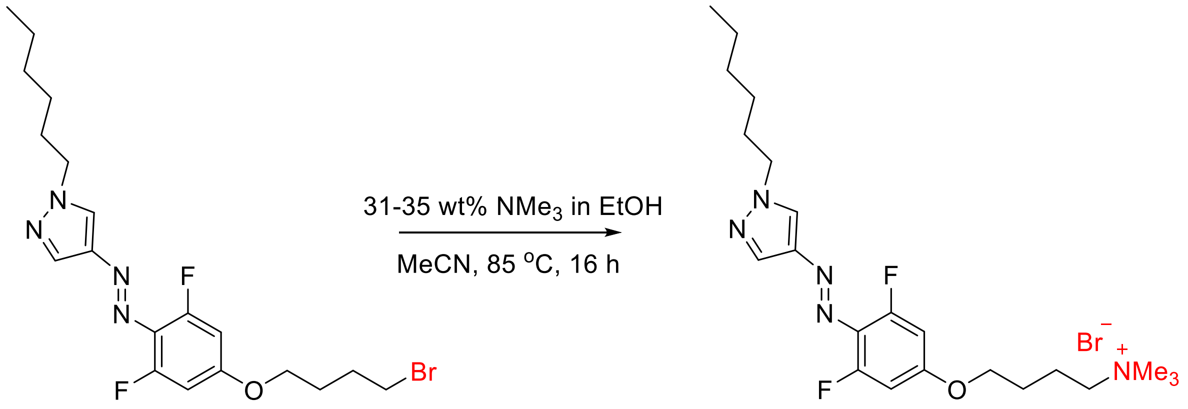

S4 **PS**

To a dried Schlenk flask was added **S4** (0.40 g, 0.9 mmol, 1 eq), 31-35 wt% trimethylamine solution in ethanol (0.25 g, 1.4 mmol, 1.5 eq) and anhydrous MeCN (20 mL), under N_2. The flask was left to heat at 85 °C for 16 h. Over this time, a precipitate formed in the flask. The yellow precipitate was collected by filtration and washed with cold Et_2O (60 mL) yielding **PS** as a yellow solid (0.42 g, 93% yield); ^1H NMR (400 MHz, 298 K, DMSO-d_6): δ 8.53 (s, 1H, ***H***-5), 7.93 (s, 1H, ***H***-3), 6.96-6.89 (m, 2H, Ar-H), 4.20-4.09 (m, 4H, NC***H***$_2$ and OC***H***$_2$), 3.51-3.41 (m, 2H, C***H***$_2$NMe$_3$), 3.11 (s, 9H, CH$_2$N***Me***$_3$), 1.92-1.70 (m, 6H, NCH$_2$C***H***$_2$ and OCH$_2$C***H***$_2$C***H***$_2$), 1.31-1.18 (m, 6H, (C***H***$_2$)$_3$CH$_3$), 0.82 (t, *J* = 6.58 Hz, 3H, C***H***$_3$) ppm; ^{13}C NMR (100 MHz, 298 K, DMSO-d_6): δ 159.71, 157.5, 154.9, 141.7, 131.8, 127.8, 124.5, 99.9, 99.6, 68.2, 64.8, 52.2, 51.9, 30.7, 29.4, 25.5, 25.2, 22.0, 19.1, 13.9 ppm; $^{19}F\{^1H\}$ NMR (378 MHz, 298 K, DMSO-d_6): δ –119.79 ppm; HRMS (ESI, CD_3CN) for [**PS**–Br$^-$]$^+$: *m/z* calcd: 422.2731; found 422.2725 (Error: 1.4 PPM).

Synthesis of PS-dT

PS-dT was synthesised in an analogous way to **PS** with the modification of 1-bromohexane being substituted for 1-bromohexane-d_{13} in the synthesis of **S1** (Scheme S1). The rest of the procedure was unchanged.

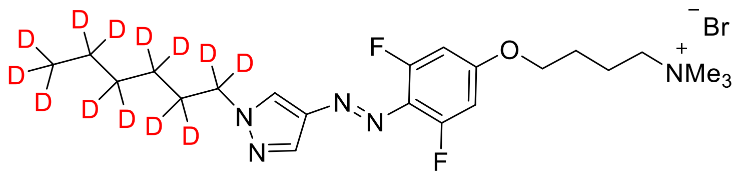

PS-dT

^{1}H NMR (400 MHz, 298 K, DMSO-d_6): δ 8.53 (s, 1H, ***H***-5), 7.93 (s, 1H, ***H***-3), 6.97-6.87 (m, 2H, Ar-H), 4.13 (t, *J* = 5.71 Hz, 2H, OC***H***$_2$), 3.43-3.35 (m, 2H, C***H***$_2$NMe$_3$), 3.08 (s, 9H, CH$_2$N***Me***$_3$), 1.91-1.71 (m, 4H, OCH$_2$C***H***$_2$C***H***$_2$), ppm; ^{13}C NMR (100 MHz, 298 K, DMSO-d_6): δ 159.71, 157.82, 154.96, 141.68, 131.80, 127.73, 124.54, 99.86, 99.60, 68.13, 64.83, 52.18, 51.83, 30.64, 29.41, 25.48, 25.21, 21.94, 19.10, 13.81 ppm; ^{19}F{^{1}H} NMR (378 MHz, 298 K, DMSO-d_6): δ –119.82 ppm; HRMS (ESI, CD_3CN) for [**PS-dT**–Br$^-$]$^+$: *m/z* calcd: 435.3547; found 435.3556 (Error: 2.1 PPM). ^{2}D NMR (400 MHz, 298 K, DMSO-d_6): δ 4.12, 1.74, 1.16, 0.74 ppm; resonances unique to ^{13}C{^{1}H} NMR (400 MHz, 298 K, DMSO-d_6): δ 51.07, 29.23, 28.18, 24.18 , 20.60, 12.65 ppm.

Synthesis of PS-dH

PS-dH was synthesised in an analogous way to **PS** with the modification of 31-35 wt% trimethylamine solution in ethanol being substituted for trimethylamine-d_9 hydrochloride along with the addition of 1 eq of NaOH in the final step of the synthesis (forming **PS** in Scheme S1).

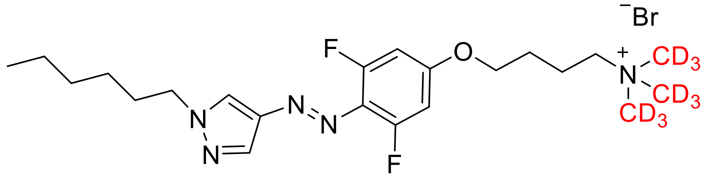

PS-dH

^{1}H NMR (400 MHz, 298 K, DMSO-d_6): δ 8.53 (s, 1H, ***H***-5), 7.93 (s, 1H, ***H***-3), 6.99-6.88 (m, 2H, Ar-H), 4.20-4.09 (m, 4H, NC***H***$_2$ and OC***H***$_2$), 3.41-3.35 (m, 2H, C***H***$_2$NMe$_3$), 1.90-

1.68 (m, 6H, $NCH_2C\boldsymbol{H}_2$ and $OCH_2C\boldsymbol{H}_2C\boldsymbol{H}_2$), 1.30-1.17 (m, 6H, $(C\boldsymbol{H}_2)_3CH_3$), 0.84 (t, J = 6.62 Hz, 3H, $C\boldsymbol{H}_3$) ppm; ^{13}C NMR (100 MHz, 298 K, DMSO-d_6): δ 159.72, 157.49, 154.89, 141.67, 131.80, 127.78, 124.49, 99.86, 99.61, 68.14, 64.53, 51.86, 51.83, 30.66, 29.41, 25.50, 25.22, 21.96, 19.04, 13.87 ppm; ^{19}F{^{1}H} NMR (378 MHz, 298 K, DMSO-d_6): δ –119.81 ppm; HRMS (ESI, CD_3CN) for [**PS-dH**–Br^-]$^+$: *m/z* calcd: 431.3296; found 431.3296 (Error: 1.2 PPM). ^{2}D NMR (400 MHz, 298 K, DMSO-d_6): δ 3.02 ppm; resonances unique to ^{13}C{^{1}H} NMR (400 MHz, 298 K, DMSO-d_6): δ 51.22

3. Supporting and Characterization data

Description of Computational Methods

DFT studies were performed using Gaussian 16.[2] The structures were initially created using Avogadro software (1.2.0) and were geometry-optimised using an MMFF94s force field.[3] DFT geometry optimisations were carried out on the MMFF94s optimised structures using the def2TZVP basis set (for the CAM-B3LYP functional) and the GD3 dispersion correction.[4] Solvation was included using a CPCM solvation model for water. Ground state structures were confirmed by checking for the absence of imaginary vibrational modes in frequency calculations performed at the same level of theory. The molecular volume of the compounds was determined by taking the average calculated volume of 15 repeat calculations (due to the procedure using a Monte-Carlo integration approach). Note that counter anions were omitted from these calculations.

Table S1. The ground state optimised structures of **CTAB**, *E*-**PS** and *Z*-**PS**. Solvation was included using a CPCM model for water (C – Grey, H – White, N – Blue, O – Red, F – Green). Note that the counter-ions were not included in the calculations. The molecular volumes shown are the average values from 15 repeat calculations and were used in SANS fitting.

Optimised Structure	Molecular Volume (cm^3/mol)
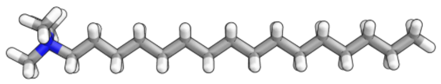	280.03
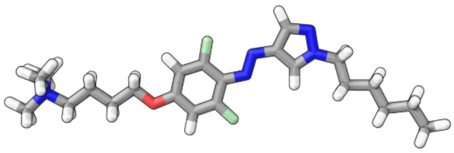	331.88
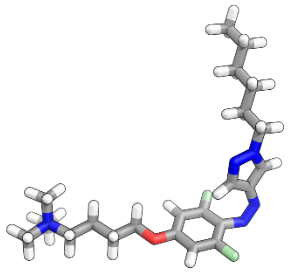	329.03

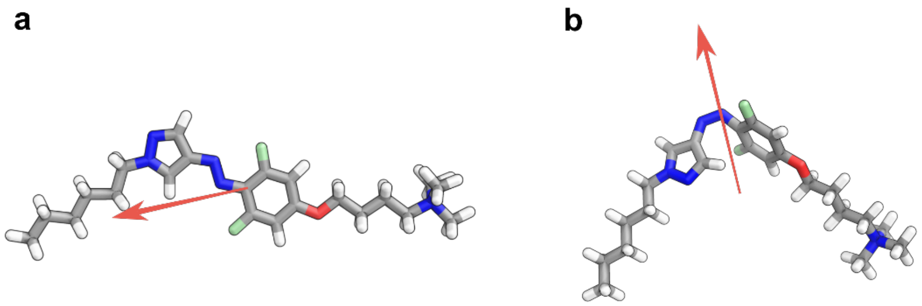

Figure S1. Structures of **a**, ***E*-PS** (*trans* state) and **b**, ***Z*-PS** (*cis* state) using the CAM-B3LYP method and the def2TZVP basis set with the GD3 dispersion correction and the CPCM solvation model for water. Red arrows indicate the direction of the dipole moment of the molecule. Note that counter anions were omitted from these calculations, the calculated dipole moment for **CTAB** (without Br^-) was determined to be 40.24 D, this large value is attributed to the naked cation. The calculated dipole moment for **a** is 49.00 and **b** is 40.21.

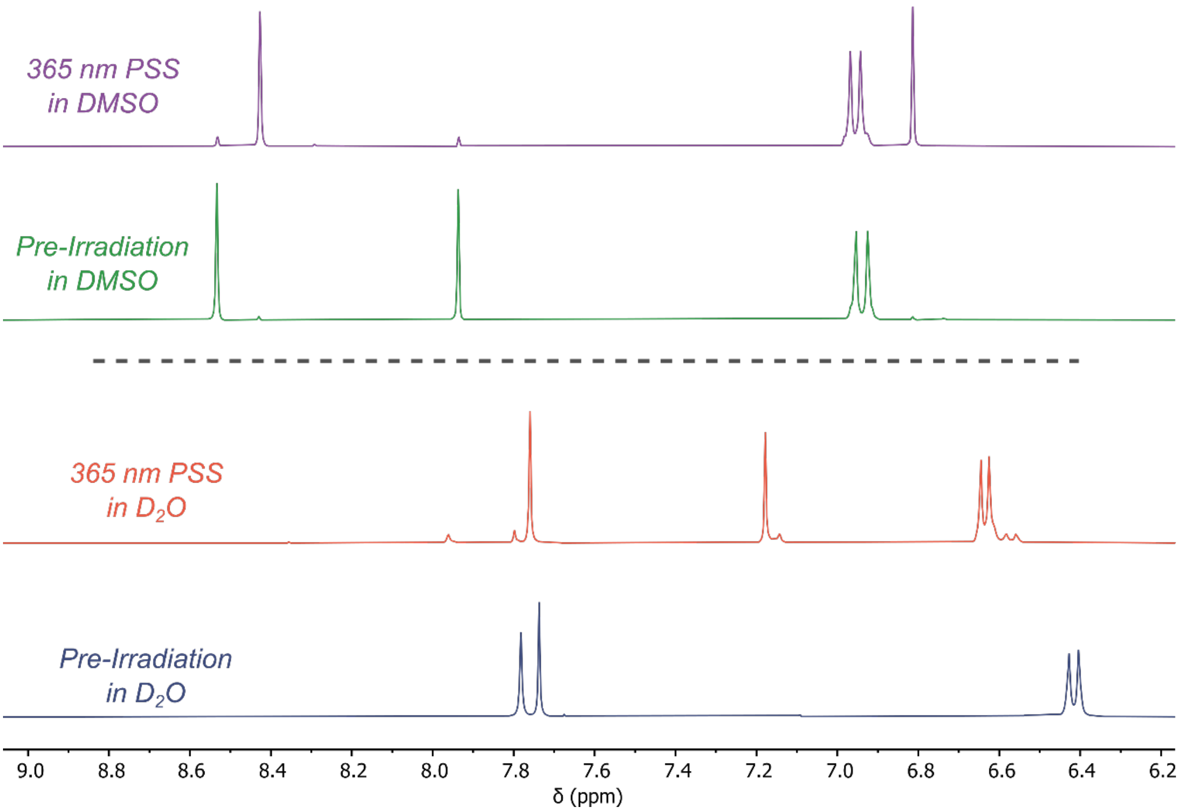

Figure S2. ^{1}H NMR spectra (400 MHz, 298 K, DMSO-D_6 or D_2O) of **PS** pre- and post-365 nm irradiation to the PSS. Samples were irradiated with 365 nm light until no further change was observed in the distribution of the *E*/*Z* isomers in the ^{1}H NMR spectra. Shifts in the *E*/*Z* signals between the blue and red trace (both in D_2O) is attributed to the formation of micelles changing the local environment of the molecules. Such differences in position of the signals were not observed when the sample was molecularly dissolved in DMSO, The 365 nm PSS in DMSO is 93% *Z* and 89% *Z* in water.

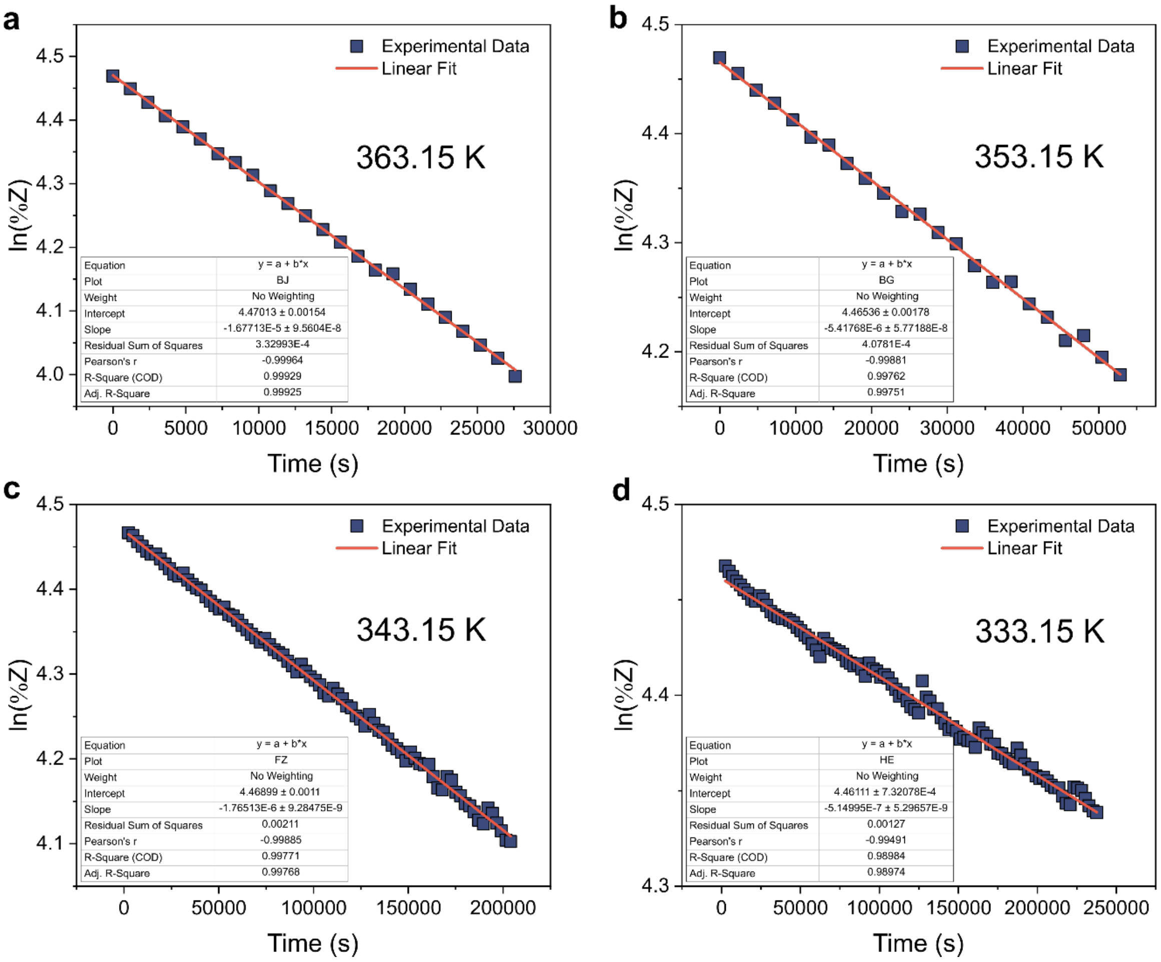

Figure S3. The percentage of the *Z*-isomer present of switch **PS** over time for temperatures, **a** 363 K, **b** 353 K, **c** 343 K and **d** 333 K. The percentage of the *Z* isomer was determined via UV-vis absorption measurements. The measurements were conducted in an air-tight cuvette, using H_2O as the solvent. The sample remained inside the instrument, in the absence of ambient light, and the temperature applied to the sample was varied. Each sample was initially converted to its 365 nm PSS in H_2O (89% *Z*). The decay of %*Z* over time at each temperature was fitted to a straight line, the R^2 value of the fit is shown in the insets. The fitted parameters were used in Figure S4 to estimate the thermal half-life of the switch at room temperature.

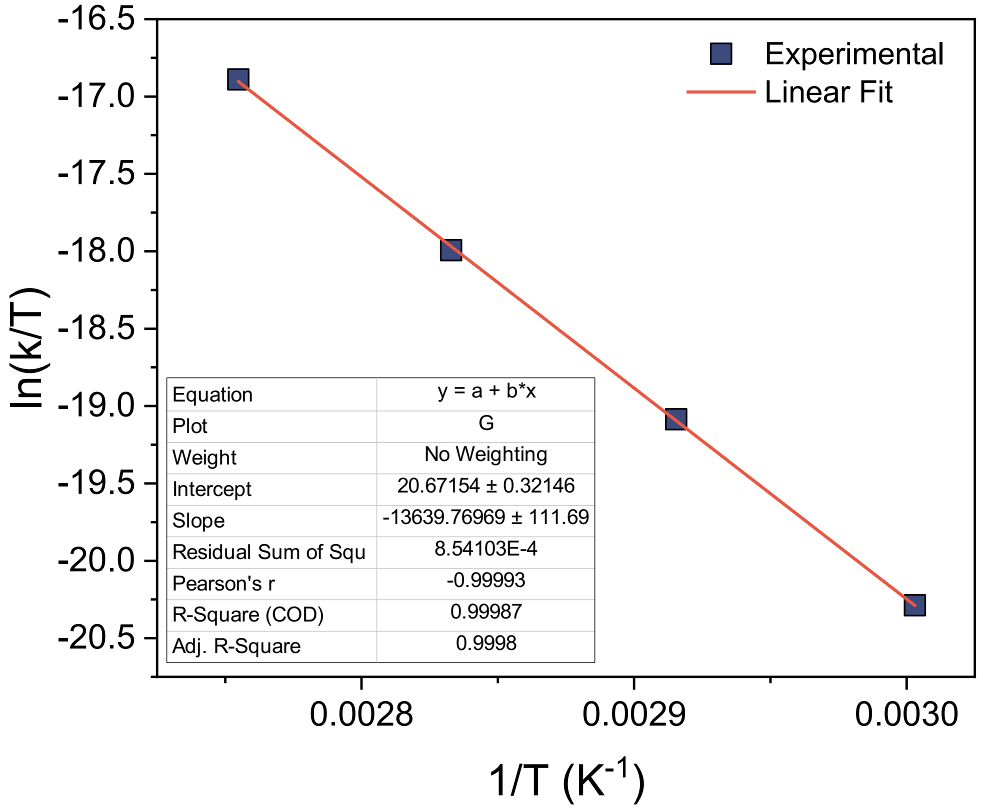

Figure S4. The Eyring plot of **PS** using the rate constants calculated in Figure S3. The data was fitted to a straight line (R^2 = 0.999), allowing the thermal half-life at 298.15 K to be determined (5.7 years in H_2O). The activation energy barrier (E_a), entropy ($\Delta S^‡$), and Gibbs free energy ($\Delta G^‡$), were calculated to be 113.4 kJ/mol, -25.7 J/(K mol), and 121.1 kJ/mol respectively.

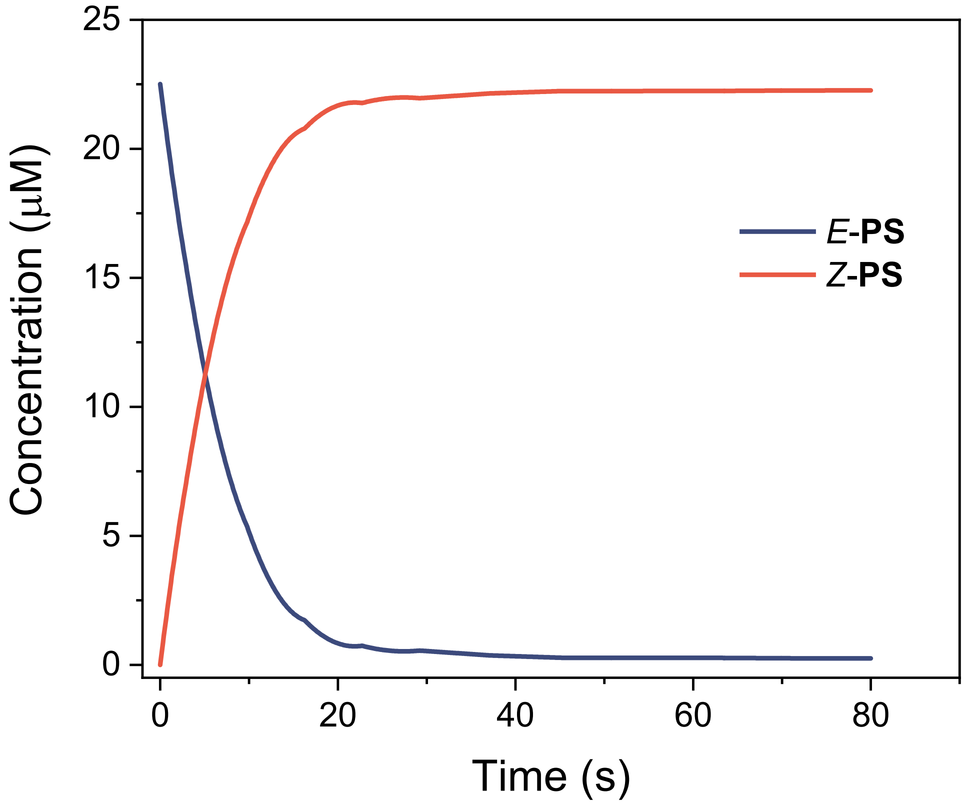

Figure. S5. Plot of concentration of switch **PS** as a function of irradiation time with 365 nm light. This was used to calculate the quantum yield of 365 nm irradiation.

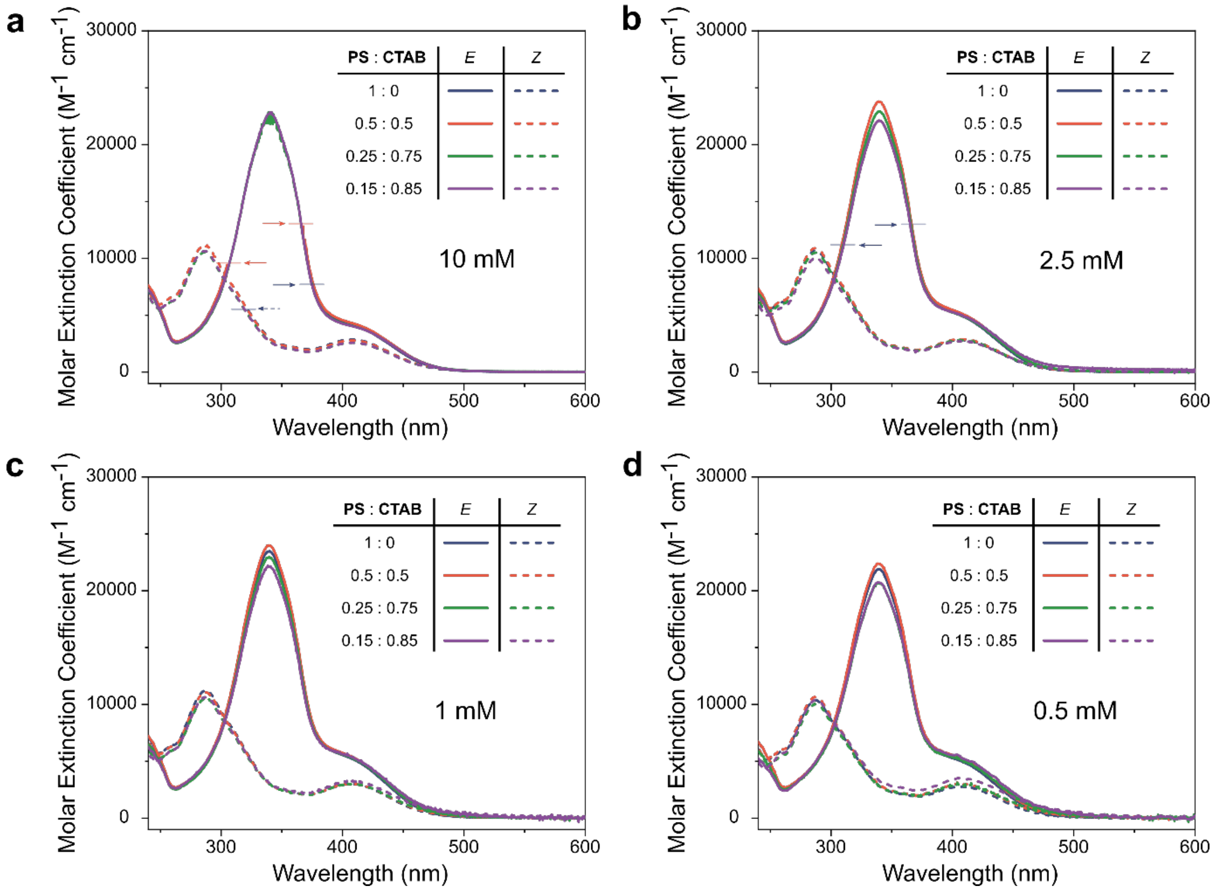

Figure S6. Plot of molar extinction coefficient of different compositions of **PS** and **CTAB** at varying solution concentrations (10 mM to 0.5 mM, 0.5 mm pathlength cuvette). The pre-irradiated state (*E*-rich) is shown as a solid line, the 365 nm PSS (*Z*-rich, 89%) of the samples is shown with dashed lines. The extinction coefficient was calculated with respect to the amount of **PS** in the sample. In **a** and **b**, the arrows correspond to regions where the absorption was too high (>2) and thus this data has been omitted.

4. Structural Characterization

Small angle neutron scattering (SANS)

SANS measurements were performed to determine the aggregated structures (micelles) formed by the pure **CTAB** and **PS** in the *E* state, which provided information on the geometry of structures achieved by introduction of photo-switch in the aliphatic surfactant tail and estimate of the CMC of the **PS**.

SANS measurements were carried out on the time-of-flight SANS2D diffractometer at ISIS pulsed neutron source (Oxfordshire, UK), with an incident wavelength range of 2 – 14 $Å^{-1}$, and wavevector Q range of 0.005 – 1 $Å^{-1}$ achieved by two detectors at 2.4 and 4 m from the sample. Banjo quartz cells of 1 mm path length were used for the measurements. The data were reduced and calibrated using MANTID, radially averaged and analysed with SasView (v5.0.4) using an ellipsoid form factor and Hayter mean spherical approximation (HMSA) structure factor. To achieve partial isomerization, for PS aqueous samples were irradiated with a wavelength of 365 nm for 20 min using the UV lamp with power of around 20 mW cm^{-2}. Isomerization was measured using UV-vis absorption spectra of samples in the diluted state.

Small angle X-ray scattering (SAXS)

The SAXS measurements were performed at BioSAXS beamline B21, Diamond Light Source (Oxfordshire, UK).[5] The X-ray beam energy was 12.4 KeV and the scattering intensity was collected at a detector distance of 4.014 m with the Q range-0.0031 – 0.34 $Å^{-1}$. Samples were loaded into a 96-well PCR plate and stored at 30°C before injection into a quartz capillary, held at 30°C, for measurement. All the samples were moved at 1 µL/s through the beam to avoid beam damage. 2D diffraction patterns were radially averaged and integrated to get 1D data. The solvent background was subtracted using ScÅtter software. To achieve full isomerization, for PS and PSM, aqueous samples were irradiated with a wavelength of 365 nm for over 6 hours using the LED lightbox with power of around 6 mW cm^{-2}. Samples were rotated every hour to ensure full isomerization. Isomerization was confirmed using UV-vis spectra of samples in the diluted state.

Dynamic light scattering (DLS)

Correlograms and size distributions were obtained from measurements on a time-resolved fiber optic DLS instrument (VASCO KIN, Cordouan Technology, Pessac, France). Approximately 5 mL of each solution was measured directly in glass vials employing a detector angle of ~170°. For each sample, the instrument laser power was tuned to maximize the measured coherence (β value) of the correlogram prior to measuring for ~30 s per sample. Intensity correlograms were analyzed via a sparse Bayesian learning, which provided the lowest residual values across the correlogram, with the instrument's NANO KIN software.

Dye loaded micelles

Nile red solution (0.9 mg/ml in acetone) was prepared and transferred into glass vials. The solvent was carefully removed leaving red thin solid films on the walls of vials. Equal volume of aqueous surfactant solutions of *E*-**PS** with a concentration of 10 mM was then added to the sample vials. The solutions were stirred for 12 h at room temperature facilitating the loading of nile red into the core of micelles. After 12h the dissolution of the dye was incomplete, and the insoluble solid residues were removed by filtration (0.45 μm PTFE). Half the volume of *E*-**PS** micellar solution was exposed to the required amount of UV- dose for isomerization. *E*-**PS** and *Z*-**PS** micellar solutions were finally investigated by fluorescence spectroscopy using Spectra Max M2 microplate reader, the excitation and emission wavelengths were set to 550 nm and 650 nm.

Cryo-TEM imaging

Attempt 1: A 5 μL droplet of sample solution was placed onto a copper TEM grid covered with a perforated carbon film in a controlled environment vitrifaction system (Vitrobot) at 25 °C and 95% humidity. The excess solution was removed via blotting with filter paper for 3 s. The sample was then placed into liquid ethane prior to storage in liquid nitrogen. The sample was examined with a Tecnai G2 Spirit Twin TEM at 120 kV and the images were captured using a CCD camera (FEI 2K Eagle Camera).

Attempt 2: Samples of PS (40 mM) were vitrified in a controlled environment using a Vitrobot Mark IV (FEI). Z-PSS samples were irradiated using UV light overnight and checked for isomerisation using UV-visible spectroscopy, prior to vitrification. In all cases, a volume (5 μL) of sample was applied to freshly glow-discharged holey carbon grids (Quantifoil R 2/2, 300 mesh Cu). The grids were blotted for 3 s at 20 °C then plunge frozen into liquid ethane using a Vitrobot Mark IV plunge freezer (FEI) at 100 % relative humidity. Screening and data acquisition was carried out on a Talos Arctica microscope operated at 200 kV (Thermo Fisher) equipped with an energy filtered (slit width 20 eV) K3 direct electron detector (Gatan).

5. Liposomes permeabilization assay[6]

Liposome preparation

Phosphatidylglycerol (PG) and phosphatidylcholine (PC) lipids were dissolved in chloroform and mixed in a 1:1 molar ratio so that the final (total) concentration of lipid is 5 mg/ml. The solvent was then evaporated while rotating the round bottom flask to create a thin film on the flask walls. Films were placed under vacuum for at least 1 h to remove excess solvent. Phospholipid films were hydrated to a 100 mM aqueous solution of calcein containing NaOH (added to facilitate the complete dissolution of calcein). The flask was swirled gently for 30 s every 5 min for 1 h to ensure all the lipids from the wall of flask fall into the solution and to achieve successful encapsulation of calcein into the core of liposomes. The calcein enclosed liposomes were subsequently extruded 21 times through a polycarbonate membrane (200 nm pore size). During the preparation and extrusion process, the liposomes were maintained above the phase transition temperature of lipids, 25°C is acceptable for 1:1 (mol/mol) PG:PC. Calcein enclosed liposomes were separated from free dye by Sephadex G-50 beads column using 1X PBS buffer. Dynamic light scattering and fluorescence spectroscopy confirmed the formation and size of liposomes, suggesting effective and consistent loading of dye into liposomes with a uniform distribution of size and an average diameter of 100 nm (Fig S13).

Dye release assay

An assay was set up with a total reaction volume of 200 μL (100 μL liposomes, 80 μL PBS buffer and 20 μL of 10 mM surfactant solution (giving final conc. of 1mM in the reaction mixture)). Assay components were added directly to the wells of a 96- well solid black plate, adding the surfactant last. The reaction volume was gently mixed, and the plate was transferred to a Spectra Max M2 microplate reader with the excitation and emission wavelengths set to 490 nm and 515 nm, respectively. Prior to the addition of surfactant, the fluorescence signal of only dye-enclosed liposomes was measured for each run and used to normalise the final data. Fluorescence data was collected every 10 s for 10 min at room temperature. To normalize the raw data, the background fluorescence of surfactants is subtracted from the fluorescence of the reaction mixture and divided by the fluorescence of dye-enclosed liposomes.

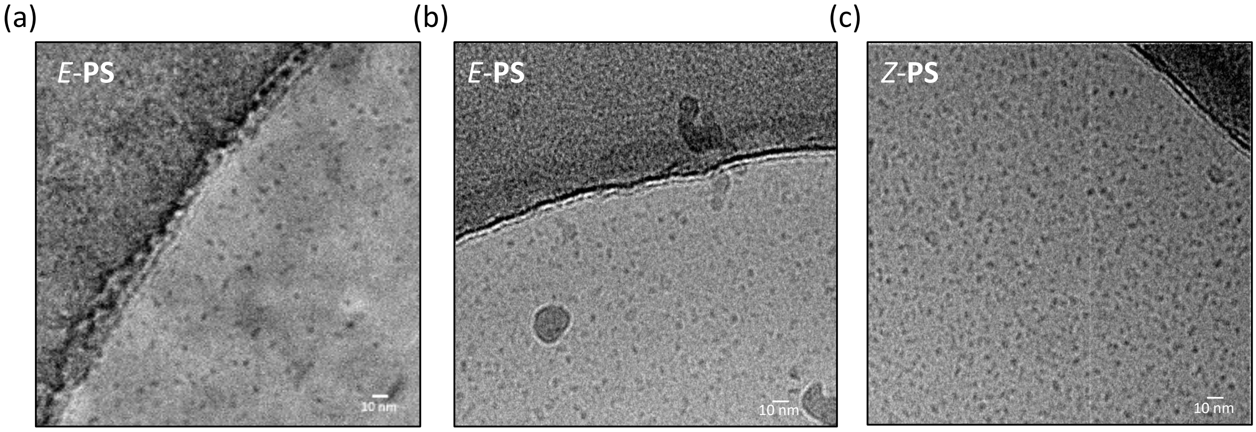

Figure S7. Cryo-TEM imaging of **a.** *E*-**PS** (8.5 mM) **b.** *E*-**PS** (40 mM) and **c.** *Z*-**PS** (40 mM) micellar solutions (quenched from 25°C) show micelles of dimensions in line with those found by SANS/SAXS. Detailed micellar dimensions and shape are extracted from scattering data, with greater discriminating power.

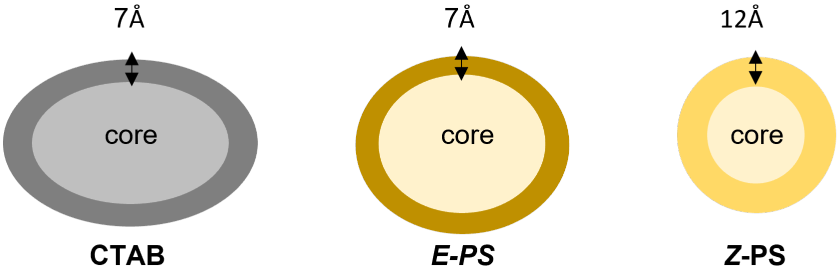

Figure S8. Schematic representing the increase in the magnitude of polar shell in case of *Z*-**PS** as compared to **CTAB** and *E*-**PS** as deduced from fitting the SAXS data.

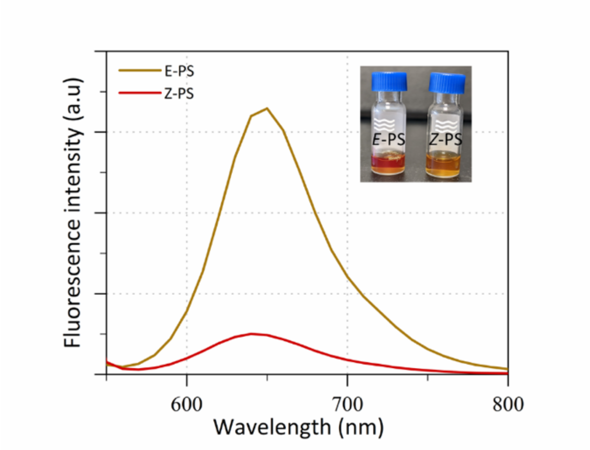

Figure S9. Fluorescence emission spectra (λ_{exc} = 550 nm) of Nile red (0.0008 mg/ml) in micellar solution of 10mM *E*-**PS** and *Z*-**PS.** A significant decrease in the emission maxima (650 nm) on isomerisation of the *E*-**PS** to *Z*-**PS** suggests that Nile red is released from the micelles into the aqueous solution on isomerisation, where the fluorescence intensity is significantly lower. The image in the inset depicts the decrease in color of micellar solution of *Z*-**PS** as compared to *E*-**PS** upon light exposure and subsequent release of dye from the micellar core.

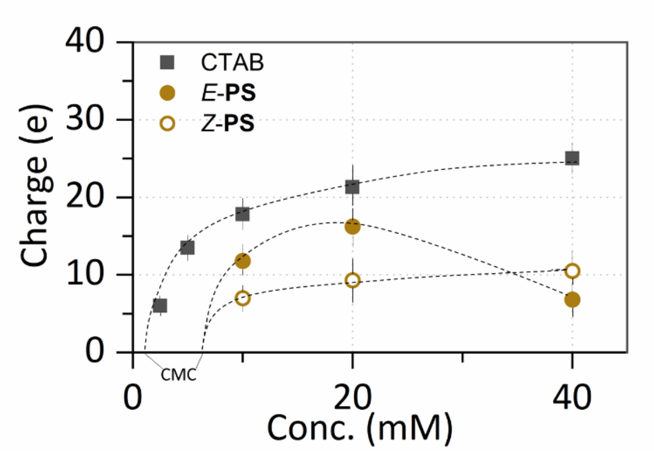

Figure S10. Fitted effective charge for **CTAB**, *E*-**PS** and *Z*-**PS** micelles as a function of concentration, estimated from the MSA structure factor model. The dashed lines are guides to the eye, starting at the approximate CMC of each surfactant. Error bars are obtained from the range of fitting parameters compatible with the scattering data. We currently do not have a definitive explanation for the decrease in charge of the *E*-**PS** at the highest concentration, obtained by fitting both SAXS and SANS data (and evident from the change in scattering profile), and we tentatively associate it with a

change in molecular packing as the micellar volume increases. *Z*-**PS** micelles exhibit a lower charge, which we associate with the conformational change of the molecules and the altered micellar shape.

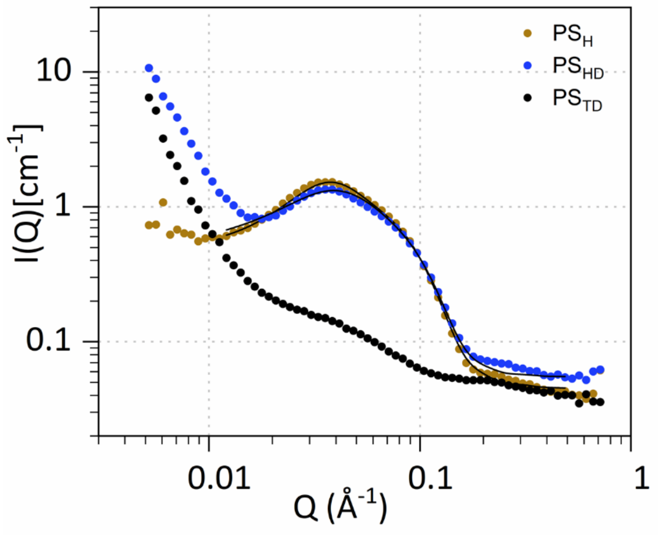

Figure S11. SANS intensity plotted against the scattering variable Q for hydrogenated (PS_H) deuterated head (PS_{HD}), deuterated tail (PS_{TD}) photo-surfactant samples in the *E*-state. The solid line represents the fit to ellipsoidal oblate model.

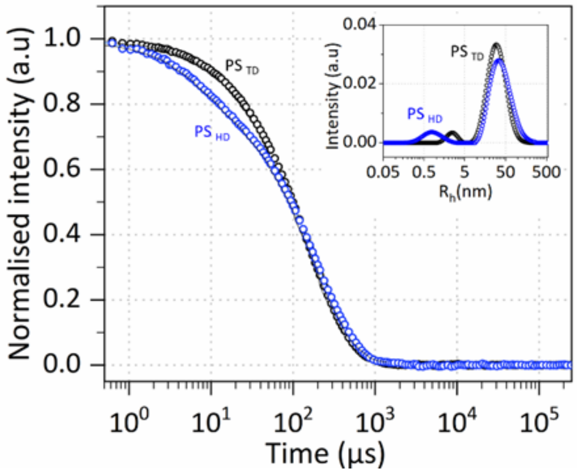

Figure S12. DLS data for micellar solutions for deuterated head (PS_{HD}), deuterated tail (PS_{TD}) photo-surfactant samples; intensity-weighted distribution of R_h for both the samples shown in inset.

Both correlograms are evident of two population of particles which is more evident in PS_{HD} then PS_{TD}. The intensity analysis of both samples shows the existence of two populations with a smaller size distribution centred around R_h=1-3 nm suggesting the presence of micelles. These results along with SANS data suggest the co-existence of micelles with a small number of large aggregates in the system. The intensity of scattered light scales with size with a large exponent and therefore large particles can contribute significantly to the scattering. Our DLS profiles do not exhibit significant influence of this and therefore we can conclude that the aggregates, albeit present, are only present in small numbers. Due to the possibility of residual contaminants in the synthesised samples, our analysis of the SANS data was restricted to the Q range (0.01 $Å^{-1}$) preceding the high scattering at low Q. The two contributions, from the mid-Q micellar scattering and the aggregate scattering at low-Q can be decoupled easily given the rapid decay (Q^{-4}) of the low-Q scattering.

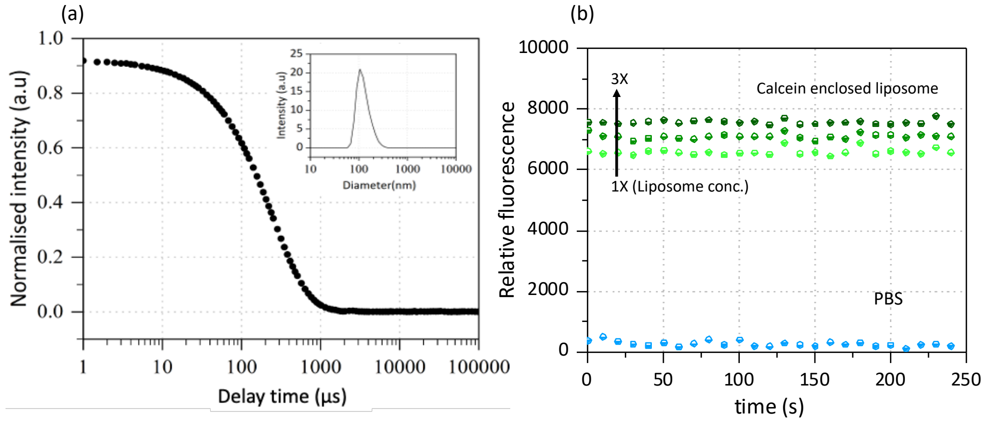

Figure S13. Characterization of liposomes via DLS and fluorescence spectroscopy. **a.** DLS data for dye (calcein) enclosed liposomes in PBS; intensity-weighted distribution of size indicating a uniform distribution of liposomes with an average diameter of 100 nm as shown in inset. **b**. Consistent fluorescence signal of calcein-enclosed liposomes prior to the addition of surfactants indicating homogeneous distribution of calcein, and efficient and uniform loading of dye into liposomes.

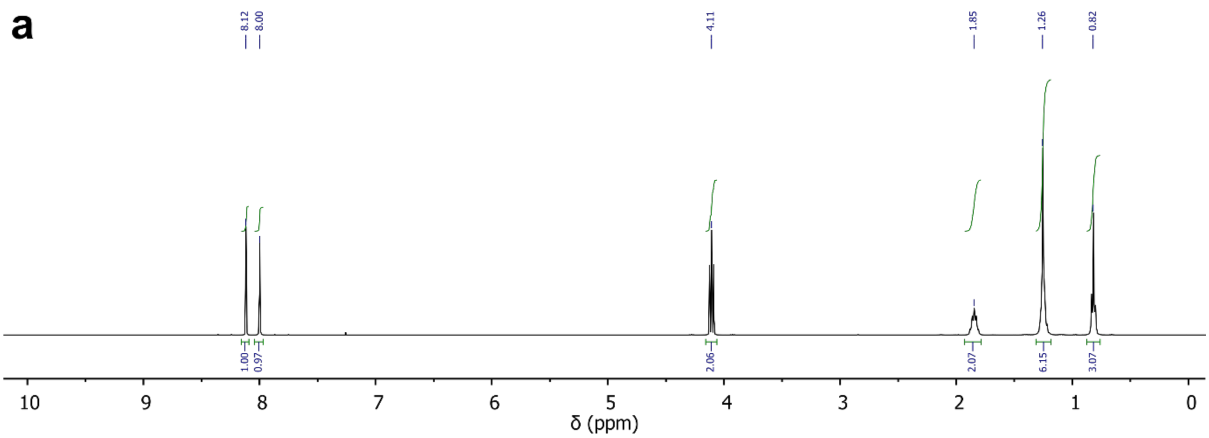

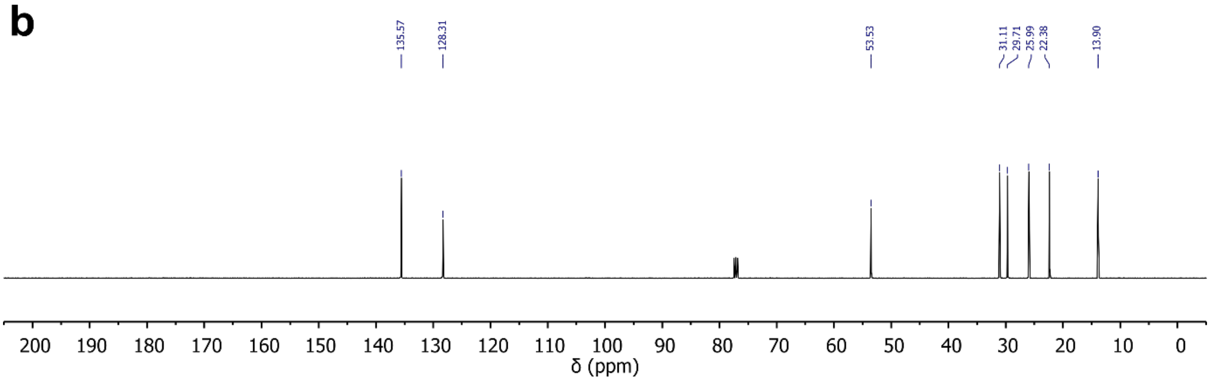

Figure S13. a, ^{1}H NMR spectrum (400 MHz, 298 K, $CDCl_3$) of **S1**. **b**, ^{13}C NMR spectrum (100 MHz, 298 K, $CDCl_3$) of **S1.**

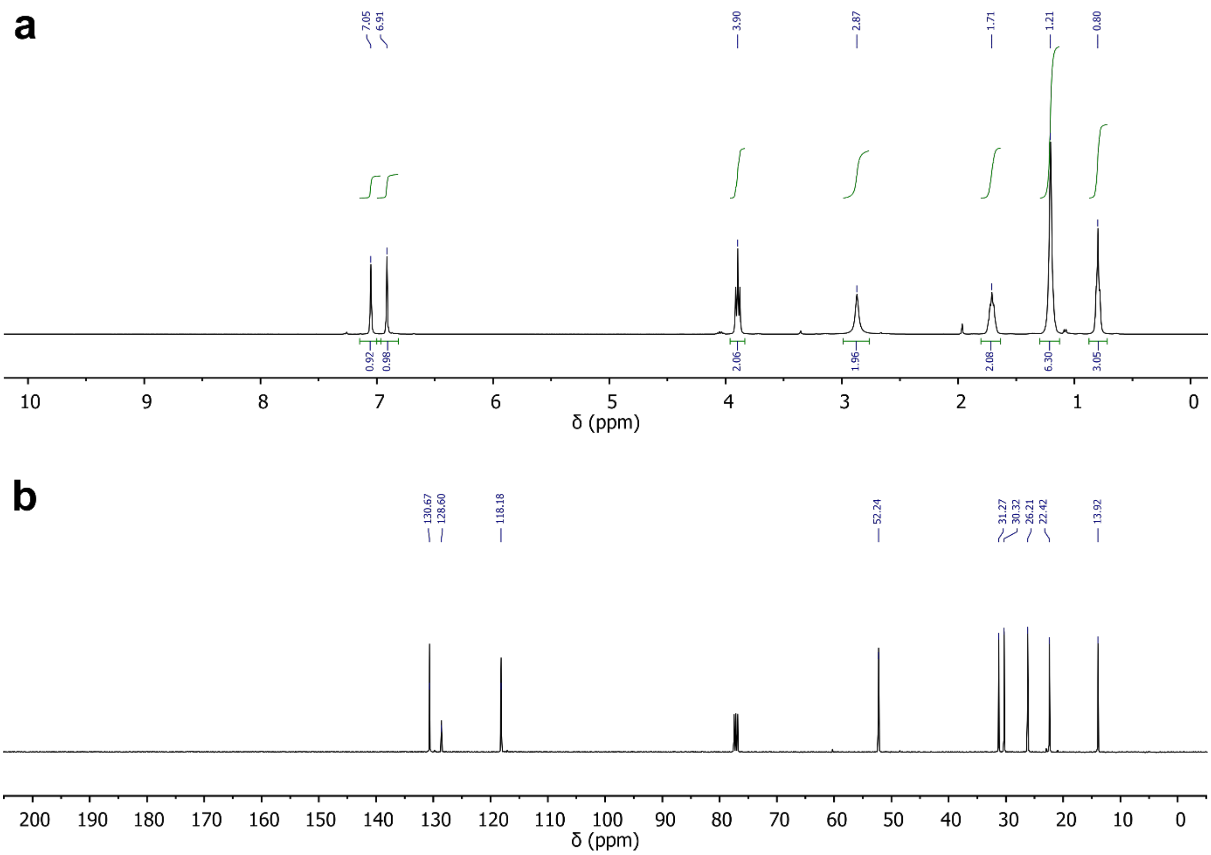

Figure S14. a, ^{1}H NMR spectrum (400 MHz, 298 K, $CDCl_3$) of **S2**. **b**, ^{13}C NMR spectrum (100 MHz, 298 K, $CDCl_3$) of **S2.**

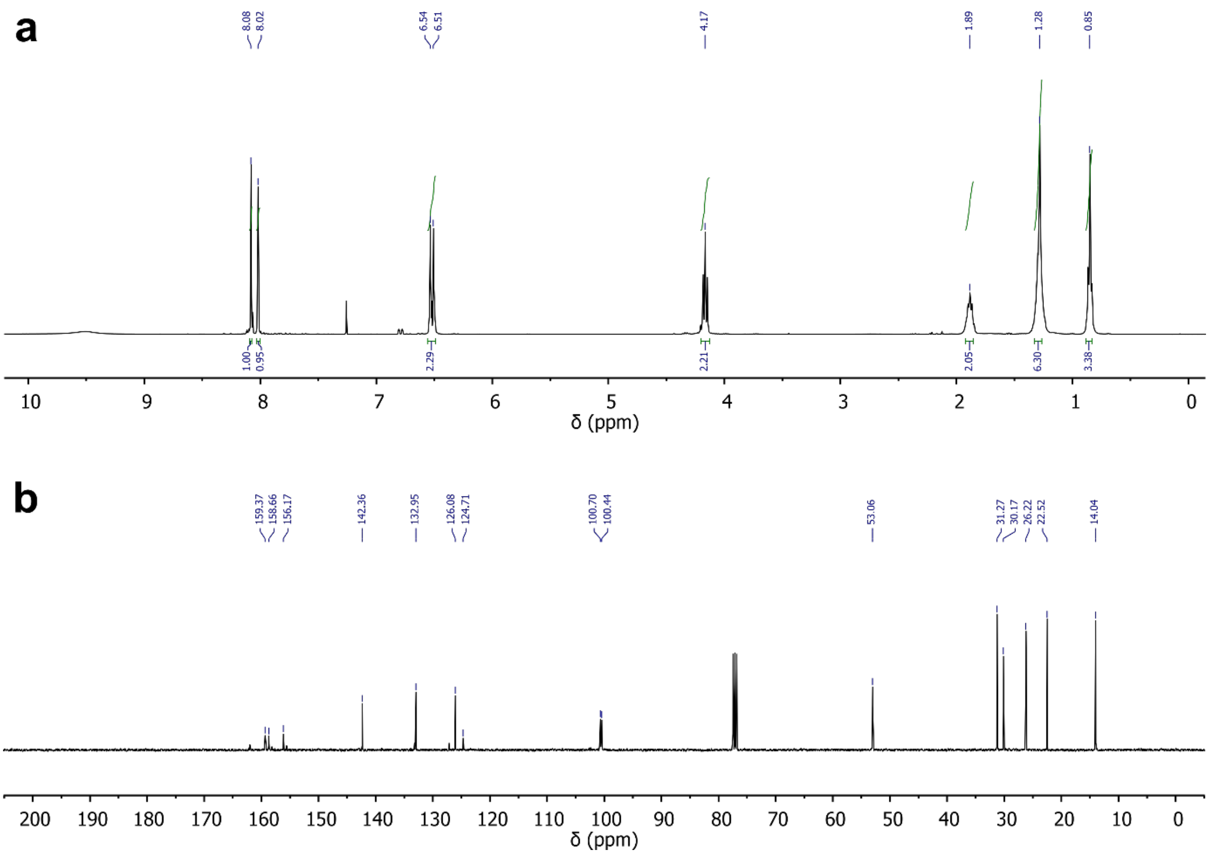

Figure S15. a, ^{1}H NMR spectrum (400 MHz, 298 K, $CDCl_3$) of **S3**. **b**, ^{13}C NMR spectrum (100 MHz, 298 K, $CDCl_3$) of **S3.**

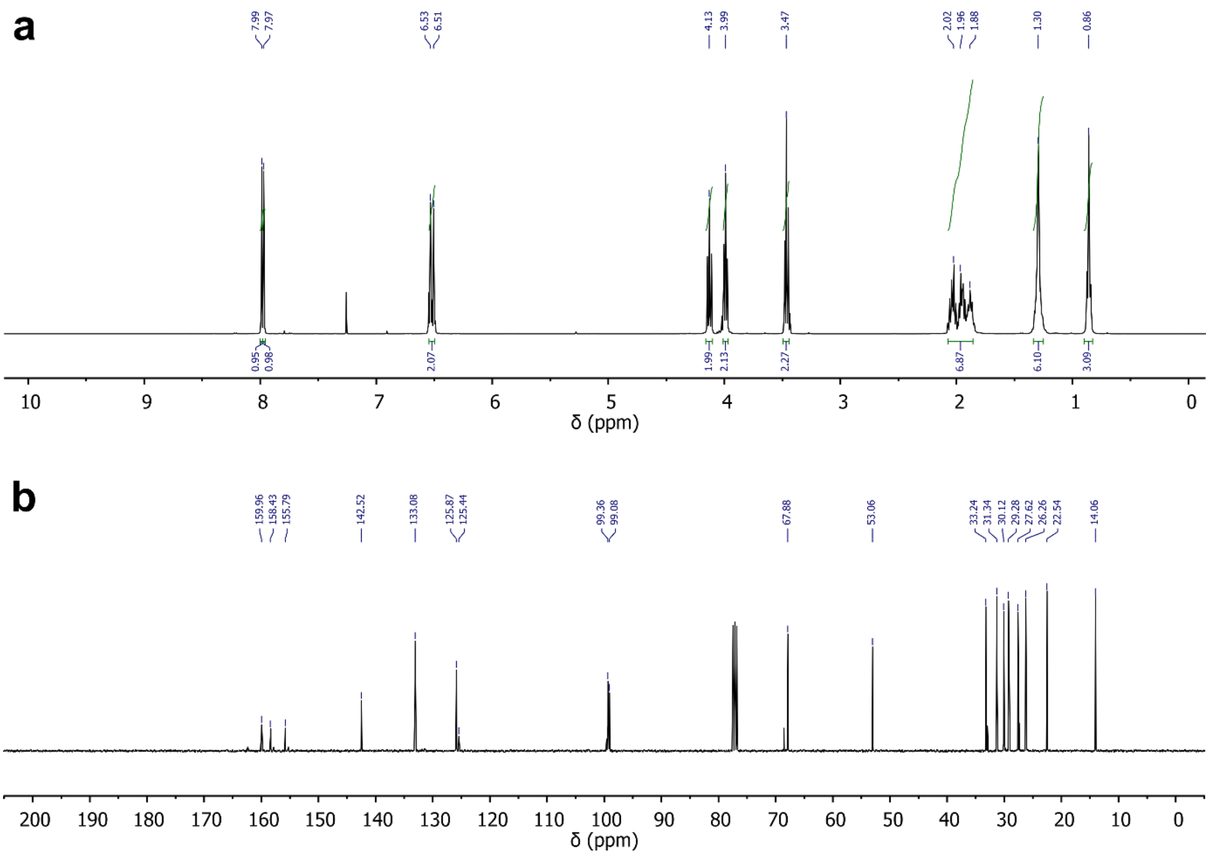

Figure S16. a, ^{1}H NMR spectrum (400 MHz, 298 K, $CDCl_3$) of **S4**. **b**, ^{13}C NMR spectrum (100 MHz, 298 K, $CDCl_3$) of **S4.**

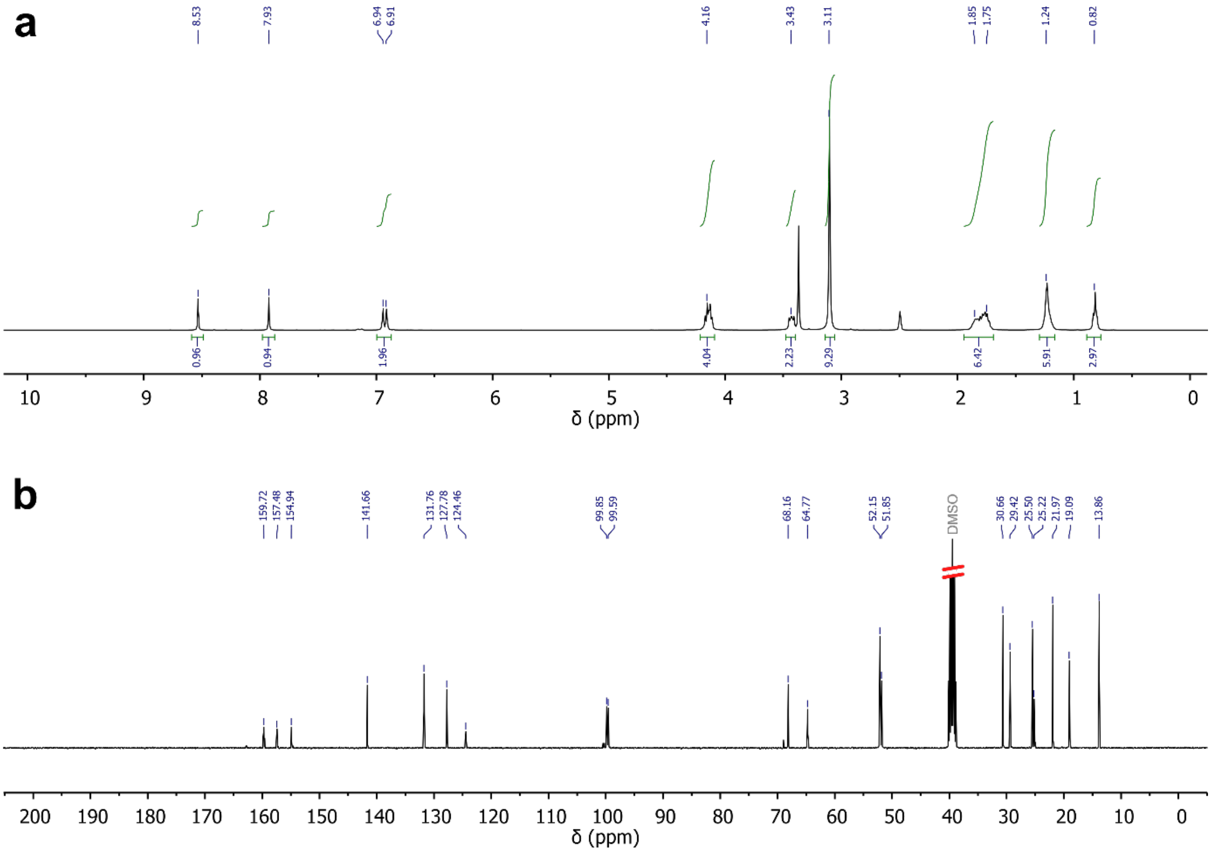

Figure S17. a, ^{1}H NMR spectrum (400 MHz, 298 K, DMSO-d6) of **PS**. **b**, ^{13}C NMR spectrum (100 MHz, 298 K, DMSO-d6) of **PS.** Note that the DMSO signal in b has been truncated (red lines).

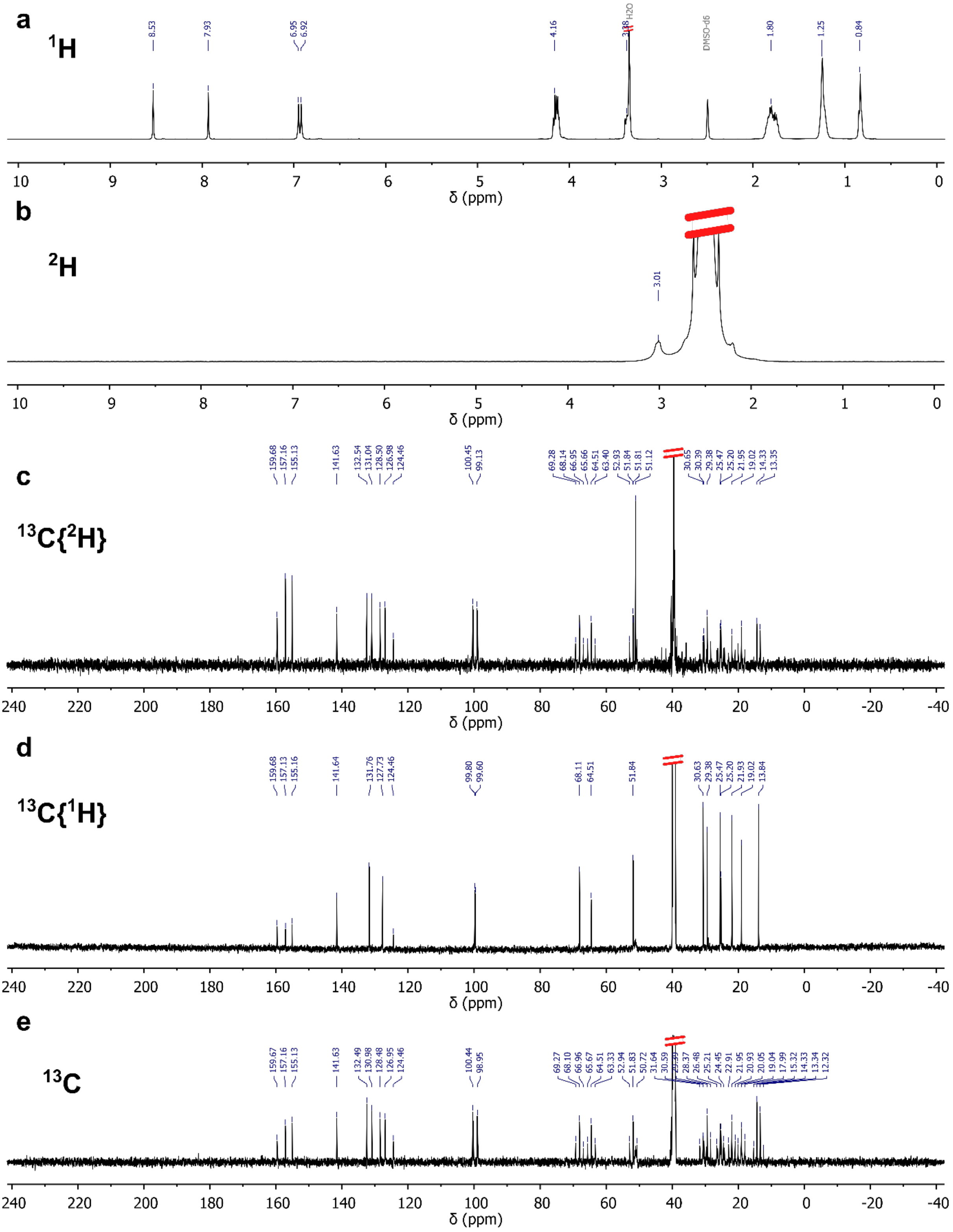

Figure S18. a, ^{1}H NMR spectrum (500 MHz, 298 K, DMSO-d6) of **PS-dH**. **b**, ^{2}H NMR spectrum (76.8 MHz, 298 K, DMSO-d6) of **PS-dH**. **c**, $^{13}C\{^{2}H\}$ NMR spectrum (125.7 MHz, 298 K, DMSO-d6) of **PS-dH**. **d**, $^{13}C\{^{1}H\}$ NMR spectrum (125.7 MHz, 298 K, DMSO-d6) of **PS-dH**. **e**, ^{13}C NMR spectrum (125.7 MHz, 298 K, DMSO-d6) of **PS-dH.** Note that the DMSO signal and residual H_2O signals have been truncated (red lines).

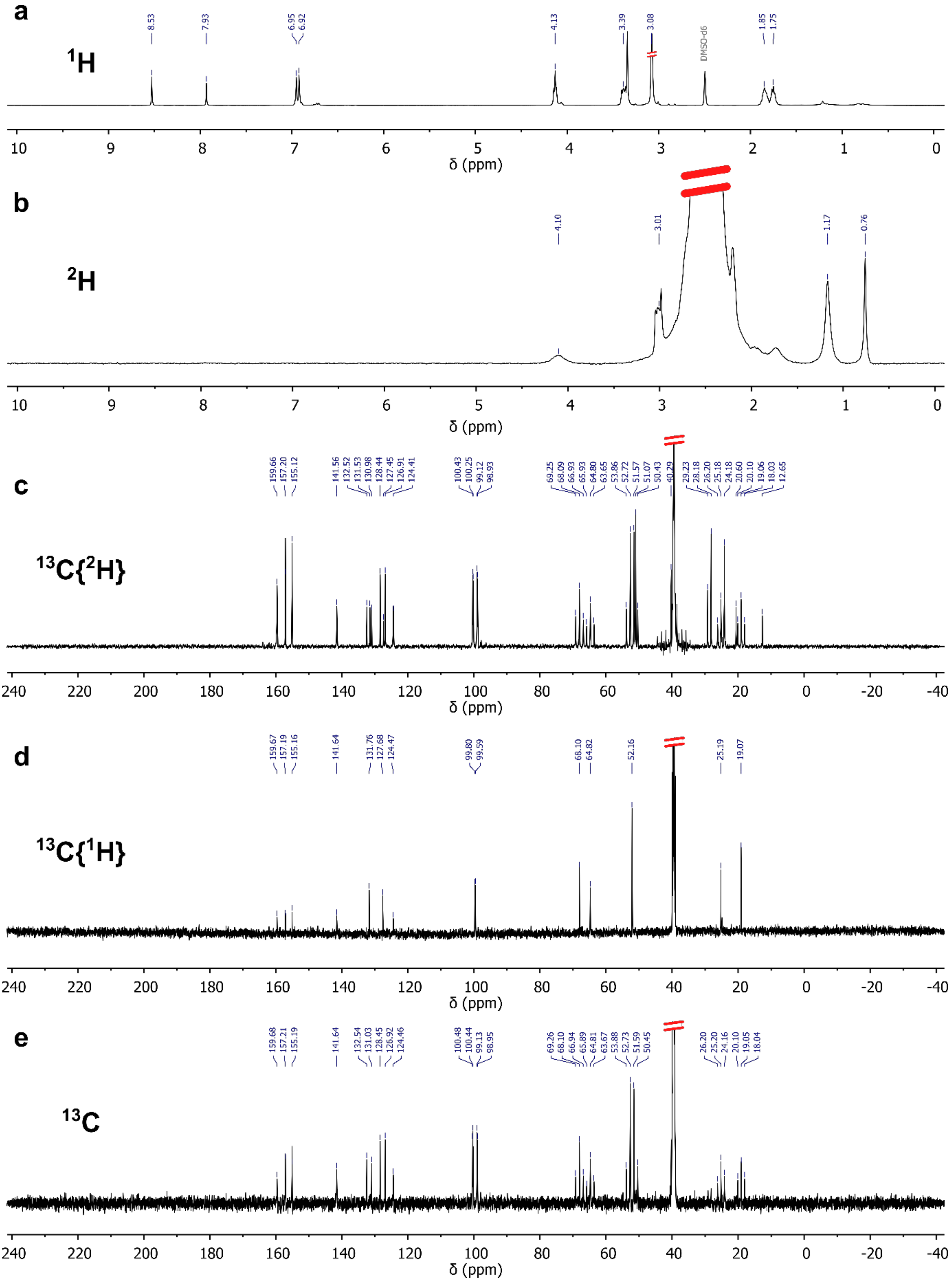

Figure S19. a, ^{1}H NMR spectrum (500 MHz, 298 K, DMSO-d6) of **PS-dT**. **b**, ^{2}H NMR spectrum (76.8 MHz, 298 K, DMSO-d6) of **PS-dT**. **c**, ^{13}C{^{2}H} NMR spectrum (125.7 MHz, 298 K, DMSO-d6) of **PS-dT**. **d**, ^{13}C{^{1}H} NMR spectrum (125.7 MHz, 298 K, DMSO-d6) of **PS-dT**. **e**, ^{13}C NMR spectrum (125.7 MHz, 298 K, DMSO-d6) of **PS-dT.** Note that the DMSO signal and the -NMe$_3$ signals have been truncated (red lines).

References

1. Stranius K, Börjesson K. Determining the Photoisomerization Quantum Yield of Photoswitchable Molecules in Solution and in the Solid State. Sci. Rep. **2017**, 7, 41145.
2. Frisch MJ, Trucks GW, Schlegel HB, Scuseria G E, Robb M A, Cheeseman J R, Scalmani G, Barone V, Petersson, GA, Nakatsuji H, Li X, Caricato M, Marenich A V, Bloino J, Janesko B G, Gomperts R, Mennucci B, Hratch DJ. Gaussian 16, Revision C.01. Gaussian, Inc.: Wallingford CT **2019**.
3. Hanwell MD, Curtis DE, Lonie DC, Vandermeersch T, Zurek E, Hutchison G R. Avogadro: An Advanced Semantic Chemical Editor, Visualization, and Analysis Platform. J. Cheminformatics **2012**, 4, 1–17.
4. Goulet-Hanssens A, Rietze C, Titov E, Abdullahu L, Grubert L, Saalfrank P, Hecht S. Hole Catalysis as a General Mechanism for Efficient and Wavelength-Independent Z → E Azobenzene Isomerization. Chem **2018**, 4, 1740–1755.
5. Cowieson NP, Edwards-Gayle CJC, Inoue K, Khunti NS, Doutch J, Williams E, Daniels S, Preece G, Krumpa NA, Sutter JP, Tully MD, Terrill NJ, Rambo RP. Beamline B21: high-throughput small-angle X-ray scattering at Diamond Light Source. J Synchrotron Radiat. **2020** Sep 1;27(Pt 5):1438-1446.
6. Dutta, S, Ben GW, Seema M, and Jean-Christophe R. Calcein release assay to measure membrane permeabilization by recombinant alpha-synuclein. Bio-protocol **2020,**10,e3690-e3690.
